# A New Photoactivatable Ruthenium(II) Complex with an Asymmetric Bis-Thiocarbohydrazone: Chemical and Biological Investigations

**DOI:** 10.3390/molecules26040939

**Published:** 2021-02-10

**Authors:** Marianna Pioli, Nicolò Orsoni, Mirco Scaccaglia, Rossella Alinovi, Silvana Pinelli, Giorgio Pelosi, Franco Bisceglie

**Affiliations:** 1 Department of Chemistry, Life Sciences and Environmental Sustainability, University of Parma, 43124 Parma, Italy; pioli.marianna@gmail.com (M.P.); nicolo.orsoni@studenti.unipr.it (N.O.); mirco.scaccaglia@unipr.it (M.S.); giorgio.pelosi@unipr.it (G.P.); 2 Department of Medicine and Surgery, University of Parma, 43126 Parma, Italy; rossella.alinovi@unipr.it (R.A.); silvana.pinelli@unipr.it (S.P.); 3 C.I.R.C.M.S.B. Consorzio Interuniversitario di Ricerca in Chimica nei Sistemi Biologici, Parma Local Unit, 43124 Parma, Italy; 4 C.O.M.T. Centre for Molecular and Translational Oncology, University of Parma, 43124 Parma, Italy

**Keywords:** thiocarbohydrazone, Ru(II) complex, photoactivation, anticancer activity, asymmetric complexes

## Abstract

The synthesis, photoactivation and biological activity of a new piano-stool Ru(II) complex is herein reported. The peculiarity of this complex is that its monodentate ligand which undergoes the photodissociation is an asymmetric bis-thiocarbohydrazone ligand that possesses a pyridine moiety binding to Ru(II) and the other moiety contains a quinoline that endows the ligand with the capacity of chelating other metal ions. In this way, upon dissociation, the ligand can be released in the form of a metal complex. In this article, the double ability of this new Ru(II) complex to photorelease the ligand and to chelate copper and nickel is explored and confirmed. The biological activity of this compound is studied in cell line A549 revealing that, after irradiation, proliferation inhibition is reached at very low half maximal inhibitory concentration (IC_50_) values. Further, biological assays reveal that the dinuclear complex containing Ni is internalized in cells.

## 1. Introduction

The term photoactivated chemotherapy (PACT) is used to define those compounds that respond to a light stimulus with a transformation of their structure [1]. The aim of this relatively new branch of biological chemistry is the synthesis of compounds that are completely inactive in cells but, once irradiated, change their structure and switch on the biological action. In this way, we can achieve control of the location, the timing, and the dosage of the therapeutic compound.

In this field, Ru(II) complexes play a relevant role. In fact, Sadler and co-workers [2,3] have developed a series of organometallic Ru(II) complexes with the general formula [(η^6^-arene)Ru-(N,N′)(L)][PF_6_]_2_, where N,N′ is a chelating ligand and L is a pyridine or pyridine-like ligand. The peculiarity of these complexes is their ability to release the monodentate ligand when irradiated with UVA light. The photodissociation and the release of the pyridine ligand induces the formation of the aqua species [(η^6^-arene)Ru(N,N′)(OH_2_)]^2+^ and this is the moiety that is effectively active inside the cell. The aim of this work is expanding and improving the original idea of Sadler and co-workers introducing a more elaborate monodentate ligand.

**Figure 1:**
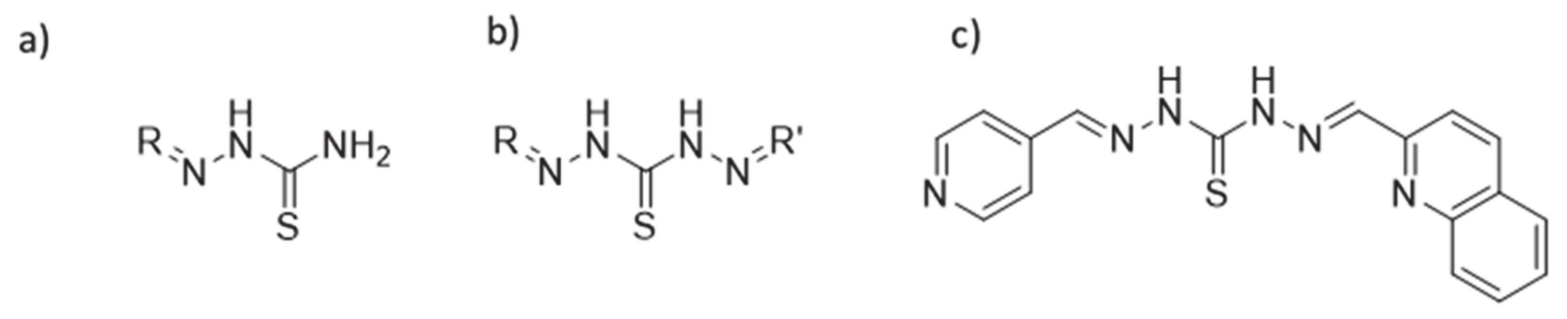
General structures of a thiosemicarbazone (**a**) and a thiocarbohydrazone (**b**) structure of the thiocarbohydrazone described in this paper (**c**). R and R′ can be both alkyl and aromatic moieties.

Our main interest has been focused on the development of ligands bearing nitrogen and sulfur as donor atoms, and on the synthesis of their metal complexes [4,5,6,7,8]. These compounds, mainly belonging to the class of thiosemicarbazones [9], are known for their wide range of biological activities and for their ability to chelate metal ions. For this reason, their behavior in solution is also remarkable [10,11,12,13,14]. Usually, the presence of the metal ions brings to an enhancement of the biological activity of the parent compounds [9]. To afford a double functionalization in the monodentate ligand, we decided to design an asymmetric thiocarbohydrazone. These kinds of compounds are already known to possess interesting biological properties [15,16,17,18], even if, unlike thiosemicarbazones, not many studies have been carried out to evaluate the biological activity of their metal complexes. In this way, the data available are few concerning the molecular basis and the biological pathways underlying their pharmacological activity [16]. This is a class of molecules very similar to thiosemicarbazones (Figure 1a), characterized by two hydrazone fragments on both sides of a thiocarbonyl group (Figure 1b). It is noteworthy that thiocarbohydrazones share an extra metal binding motif with respect to thiosemicarbazones, so they can potentially form both mononuclear and dinuclear complexes upon metal binding. Thiosemicarbazones have attracted relevant interest as anticancer agents in recent years. They have a remarkable antiproliferative activity initially ascribed to their metal sequestration ability mostly leading to the inactivation of ribonucleotide reductase (RNR), the enzyme converting ribonucleotides into deoxyribonucleotides and whose activity correlates with cell proliferation. Moreover, additional targets have emerged from more recent studies, including topoisomerase II, the metalloenzymes xanthine oxidase and tyrosinase, and the mitochondria signaling pathway [5]. Again, Ru-thiosemicarbazone metal complexes and half-sandwich type organometallic complexes are of particular interest because of their peculiar biological activity [19,20,21,22].

Following our idea, the thiocarbohydrazone modification allows the introduction of a moiety for the complexation of ruthenium, the pyridine ring, and a second free moiety, the quinoline, able to bind metal ions (Figure 1c).

The work here presented is relative to the synthesis and characterization of a new organometallic photoactivatable ruthenium(II) complex with an asymmetric bis-thiocarbohydrazone as a monodentate ligand. Moreover, to our knowledge, there are few cases reported in the literature concerning thiocarbohydrazone Ru complexes [23,24,25]. Herein, we have studied its chemical behavior in the dark and under photoirradiation. Subsequently, we investigated the ability of this ruthenium complex to chelate other metal ions, through the free quinoline moiety, by means of UV–visible titrations. The first row metal ions chosen for this experiment are Ni(II) and Cu(II). These results are then compared with similar titrations performed with the ligand alone, whose complexation ability is well known. Finally, the biological activity of the synthesized complex is studied towards the A549 cell line, representative of solid lung tumor. The half maximal inhibitory concentration (IC_50_) is evaluated in different conditions: in the dark, after 3 h of irradiation, with and without copper and nickel.

## 2. Results and Discussion

### 2.1. Synthetic Approach to Bond a Thiocarbohydrazone (TC) to a Ruthenium(II) Half-Sandwich

**Scheme 1:**
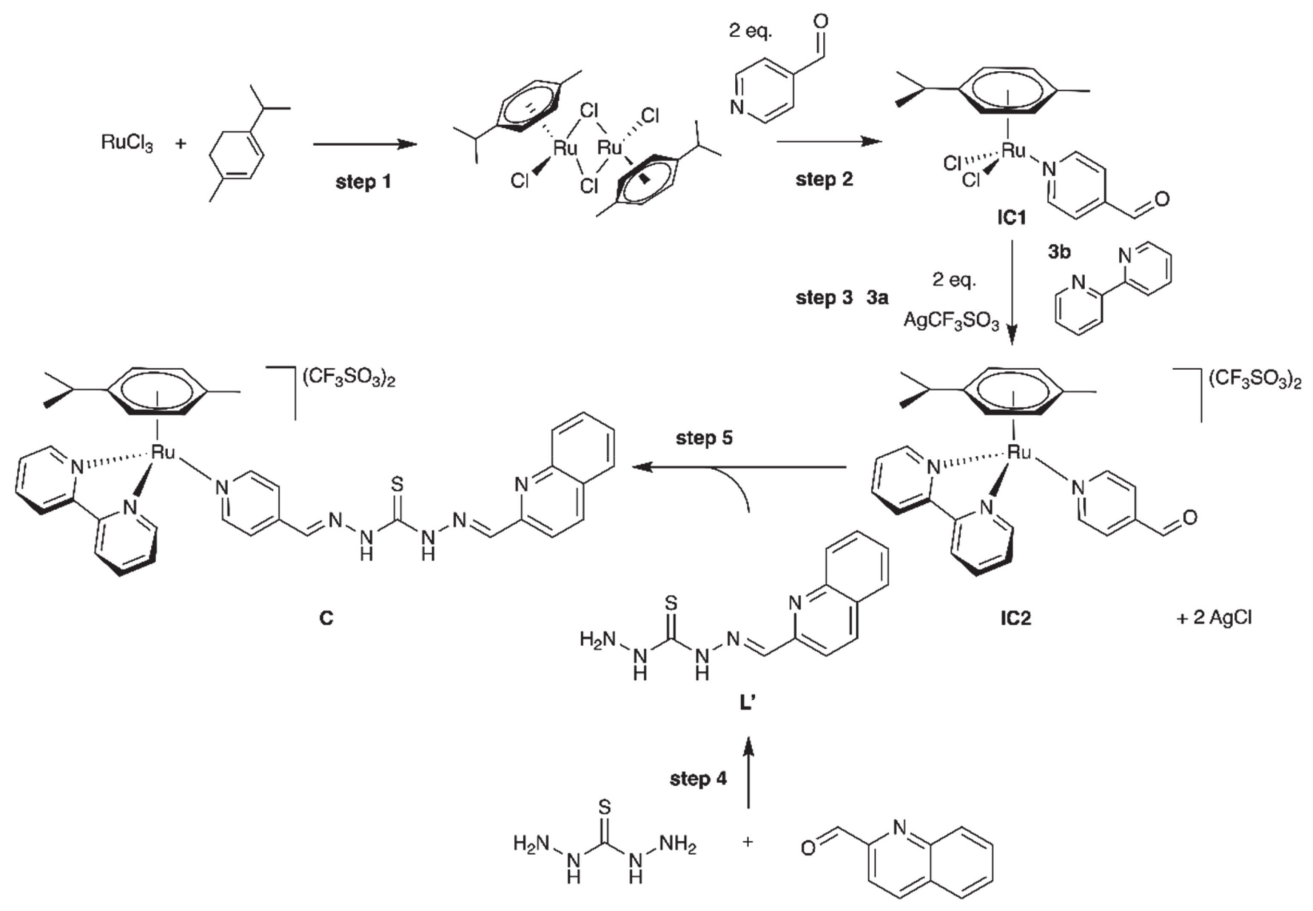
General scheme for the synthesis of the ruthenium(II) half-sandwich-thiocarbohydrazone (TC) complex.

To obtain the final product we adopted a multistep synthetic approach optimized using a convergent synthesis, described in Scheme 1.

The first step was the synthesis of the bi-metallic complex [(η^6^-p-cym)RuCl_2_]_2_. The product was obtained pure following an already known protocol which applied microwaves to speed up and maximize the reaction yield [26]. The second step was the synthesis of the [(η^6^-p-cym)Ru(4-formylpyridine)Cl_2_] complex. This step was performed by mixing two equivalents of 4-formylpyridine with one equivalent of [(η^6^-p-cym)RuCl_2_]_2_ in dry conditions to obtain two equivalents of the half-sandwich product. The subsequent step (3a) was the halogen scavenging. The neutral complex was dissolved, and two equivalents of silver triflate were added to get rid of the chlorides through the formation of AgCl. The salt formed was removed by filtration. This step was performed keeping the solution in the dark to avoid the silver photo-reduction. This step was followed by the insertion of the bipyridine (step 3b). The last step was the addition of one equivalent of bipyridine to obtain the final product. Even in this step the solution was kept in the dark to preserve the photoactivatable products. The compounds obtained were bi-cationic complexes in which two triflate anions neutralized the charges without interfering with the metal coordination. The procedure followed with the synthesis of the mono-TC ligand (step 4). The mono-TC ligand was obtained by condensation of an equimolar amount of aldehyde and thiocarbohydrazide. Finally, there was the condensation with the carbonyl group of 4-formylpyridine (step 5). The final compound was obtained from the condensation between the carbonyl group of the Ru(II) complex and the hydrazine of the TC in the dark. Routinely, the synthesis of the TC is made by refluxing the reactant mixture in methanol for 6–8 h. In this case it was necessary to use milder conditions to avoid the degradation of the complex. In the optimized protocol, we used a poorly coordinating solvent such as dry THF instead of methanol and we did not heat the mixture during the reaction. These changes in the protocol led to longer reaction times (24–48 h), but they allowed us to obtain pure products and in good yields.

### 2.2. Study of Complex Stability under Physiological Conditions

One of the key characteristics of the PACT compounds is the stability and the absence of biological activity in the dark, i.e., without irradiation. When we introduce the PACT compound in a biological medium, we must be sure that its chemical structure does not change and that its biological activity is triggered by light.

Generally, the compound is dissolved in a stock solution of DMSO (10 mM concentration) and diluted with the cell medium at the desired final concentration. Usually, the concentration of DMSO must be lower than 2%, because high concentrations can be lethal to the cells.

To study the stability of the complex, we diluted a stock solution of the complex, dissolved in DMSO, in an aqueous solution of phosphate buffered saline (PBS) characterized by a pH, an osmotic concentration of salts equal to the one found in the cell environment. The stability is evaluated comparing the UV spectrum of the complex immediately after the preparation, with the same solution after 24 h of incubation. The solution was maintained in the dark at the temperature of 37.5 °C. In Figure S1, the spectra are reported, and the stability of this complex is confirmed. The ability of the complex to absorb light in the visible region, precisely in the blue region, is also confirmed.

### 2.3. Photoactivation Studies

Once we confirmed the stability of the complex under physiological conditions in the dark, we studied its behavior when irradiated by a blue LED lamp, centered at 420 nm. The experiments were performed using a photo-oven (Figure S2) that we built in the lab. The light intensity, measured in the configuration described in Figure S2, is 405 μW/cm^2^.

The photo-oven consists of a plastic box with a light source inside which can be controlled from the outside through a switch. A lid ensures that the box is completely dark when the light inside is switched off. The sample can be placed from 0 to 25 cm from the light source and the temperature inside is controlled by a thermometer. No temperature modifications induced by the light source were registered during the experiments.

**Scheme 2:**
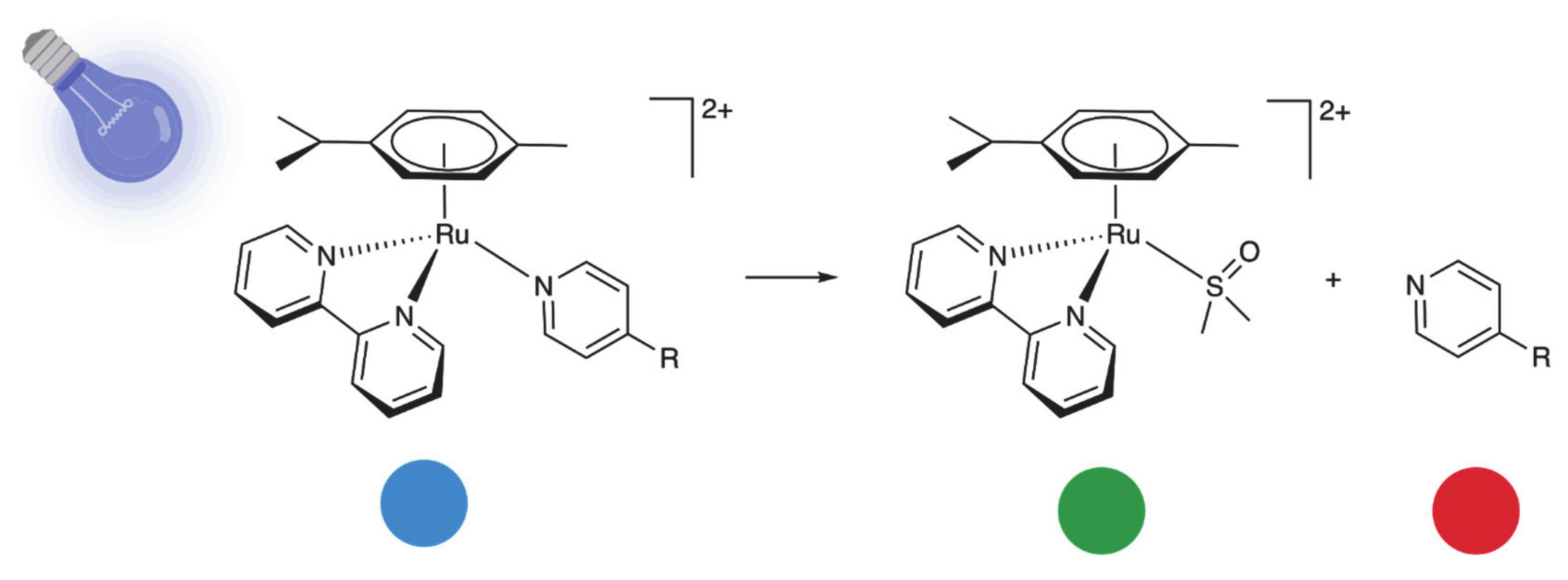
Mechanism of photolysis of the complexes.

**Figure 2:**
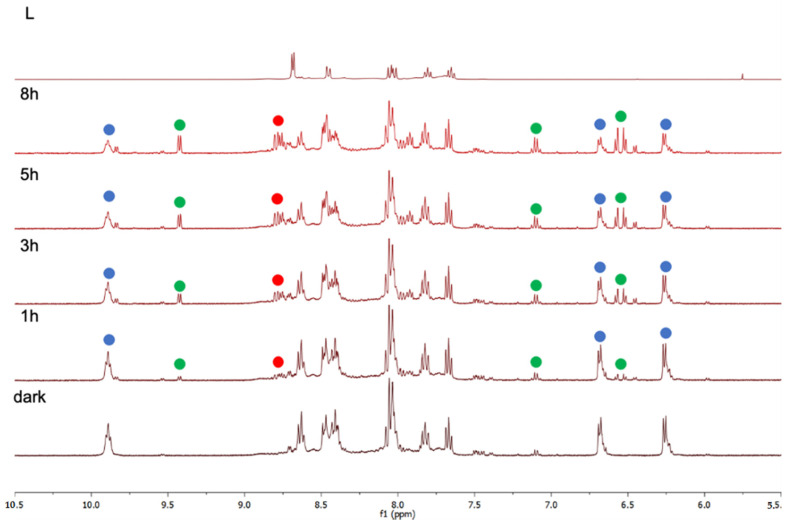
Evolution of NMR spectra of complex C after 1, 3, 5 and 8 h of photoactivation and pure free ligand L for comparison.

All the photoactivation experiments were performed on the sample contained in an NMR glass tube using a volume of 500 μL of DMSO-D6 and a concentration of 3 mM. The sample was irradiated in the NMR tube which was placed in the photo-oven at a distance of 25 cm from the light source. The effects of the light irradiation were analyzed by collecting five different spectra of the same sample. The spectrum in dark conditions was collected immediately after the sample preparation. The following spectra were collected after 1, 3, 5 and 8 h of irradiation. The results are reported by stacking the spectra in ascending order of time (Figure 2). In the same figure, the spectra of compound L are also reported for comparison. In Scheme 2, the proposed mechanism of photolysis induced by the blue light is reported. The colors of Scheme 2 are used to describe the different species in the NMR spectra in Figure 2.

In the NMR spectra reported in Figure 2, it is possible to observe that compound [(p-cym)Ru(N,N′-bpy)(N-4-formylpyridine-2-quinolinecarboxaldehydethiocarbohydrazone)](SO_3_CF_3_)_2_ (complex C) is able to change its structure upon irradiation with blue light. In fact, with the irradiation, the signals of the starting complex (blue spots, Figure 2) reduce their intensity and simultaneously new sets of signals increase their intensity. These signals are associated with the two products of photolysis: the free ligand (red spot, Figure 2) and the [(η^6^-p-cym)Ru(N,N-bipy)(DMSO)]^2+^ (green spots, Figure 2).

After 3 h of irradiation, the photorelease of the components, the free ligand and the solvate complex, is noticeable. The signals associated to the free ligand (red spot, Figure 2 and Scheme 2) are difficult to recognize because all the protons of the TC are compressed in the aromatic region, making the portion of spectra between 9.0 and 7.5 ppm extremely chaotic. Instead, the signals of the solvate complex (green spots, Figure 2 and Scheme 2) are more recognizable, especially the doublets close to 6.5 ppm, the signals of the aromatic p-cymene, and the doublet close to 9.5 ppm, linked to the complexed bipyridine. The intensity of these photoreleased products increases proportionally alongside the irradiation. In Figure S7, the comparison of the ^1^H-NMR spectrum of the complex C after 8 h of irradiation with the spectra of the pure [(η^6^-p-cym)Ru(N,N-bipy)(DMSO)] and the pure free pro-ligand is reported.

### 2.4. Studies of Complexation with First Row Transition Metals: The Case of Copper and Nickel

As described in the introduction, the thiocarbohydrazone monodentate ligand is designed to have a moiety for binding ruthenium, the pyridine, and the other end, the quinoline, necessary for the complexation of other metal ions. The quinoline moiety is formally free and not involved in any coordination. For this reason, it is necessary to explore the complexation behavior of the quinoline, with respect to metal ions, when it is linked to the ruthenium through the thiocarbohydrazone moiety.

**Figure 3:**
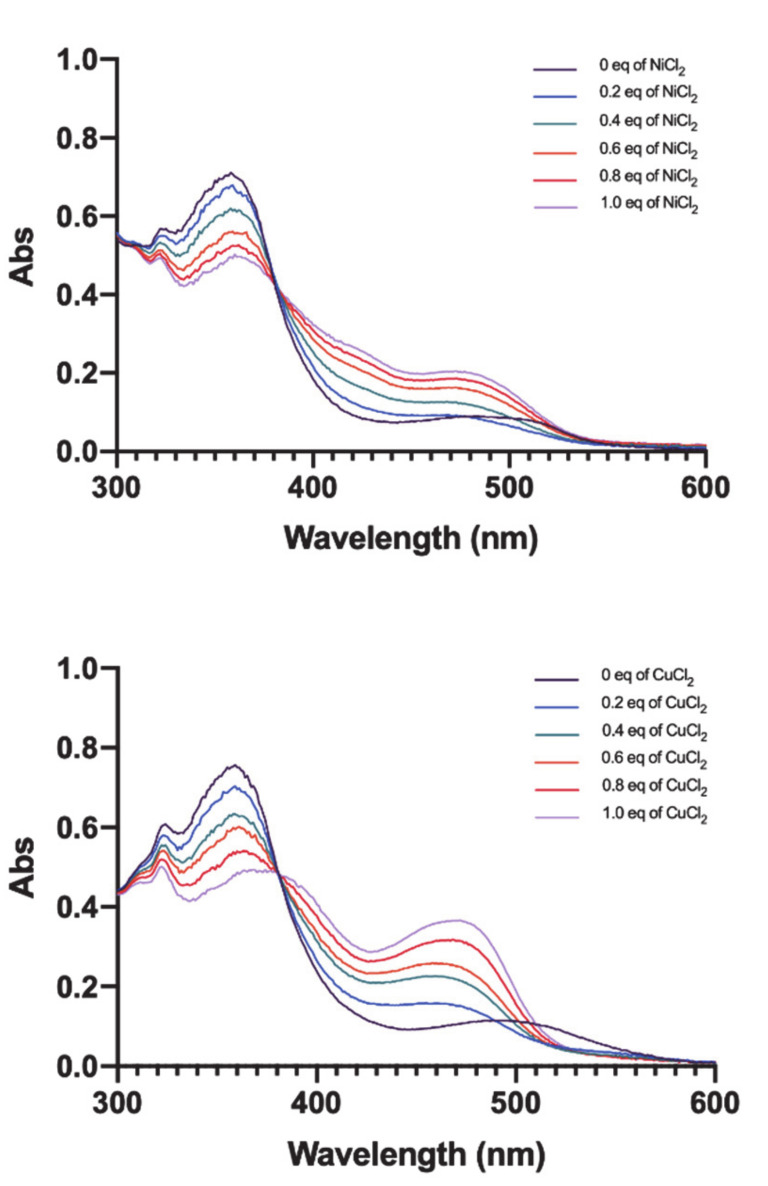
UV–vis titrations of C with nickel(II) chloride (top) and copper(II) chloride (bottom).

A series of UV–visible titrations of a solution of C with CuCl_2_ and NiCl_2_ solutions have been performed (Figure 3). These spectra are compared with the data of ligand L′ alone, whose complexation ability towards these two metals is known. The aim of these experiments is to understand if complex C is able to chelate metal ions in solution like the ligand L′ alone.

The titration was made in a quartz cuvette filled with 600 μL of a 4.85 μM solution of C in which one equivalent of the desired metal salt was added in five portions (0.2 eq. each). We used metal salt solutions more than three orders of magnitude more concentrated than C to avoid dilution interferences ([CuCl_2_] = [NiCl_2_] = 2.92 mM). The UV–vis spectra were recorded after every addition, but it was extremely important to wait for the end of the complexation before recording the spectrum. This precaution was necessary because during the first attempts we noticed that the spectra we registered immediately after the metal addition were time-dependent and non reproducible. We estimated the proper time to wait by measuring the time necessary to stabilize the UV–vis profile after a metal salt addition; this resulted in times of 7 and 30 min for copper and nickel, respectively. In addition, we registered the absorption spectra of the 4.85 μM solutions of the metal salts to verify that they would not have any absorption band which could interfere with the titration experiment.

The two titrations showed a very similar result: the gradual formation of an absorption band between 450 and 500 nm associated with the decrease in the absorption bands below 380 nm. In both cases, these changes created an isosbestic point which clearly indicated that the initial species (C) gradually converted in another single species (the hetero-dinuclear complex with copper or nickel, respectively).

**Figure 4:**
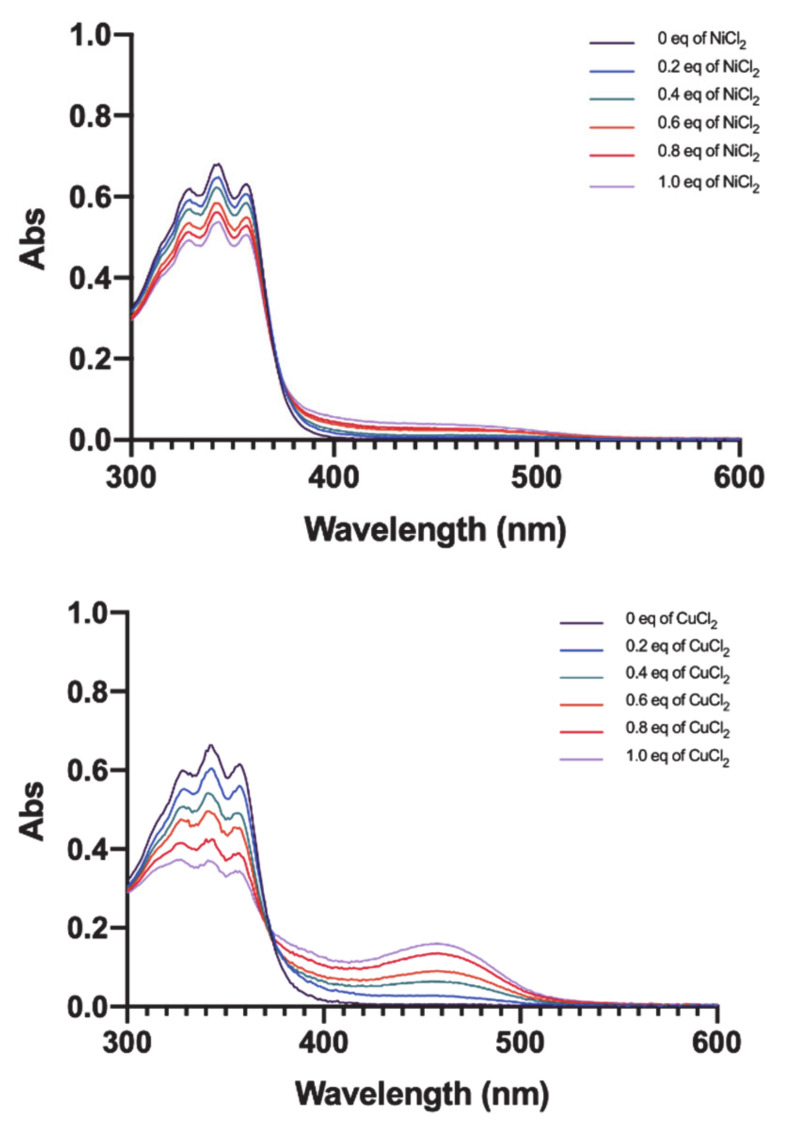
UV–vis titrations of L′ with nickel(II) chloride (top) and copper(II) chloride (bottom).

To prove that metals were κ^3^-coordinated by the quinoline-TS moiety, we performed a titration using L′ instead of C (Figure 4). L′ can interact with metals just as an S,N,N-tridentate ligand as observed in previous studies [27]. In Figure S8, the suggested structure of the hetero-dinuclear complex is reported. Therefore, the use of L′ is very useful to simplify the system and to collect information on the “quinolineTS + metal” system and the wavelengths associated with the complex formation.

Even in these spectra, the addition of both metals led to the formation of isosbestic points. This evidence confirmed once again that the species involved are linearly related by a stoichiometry process which, in this case, we could certainly assume was the formation of the complexes between L′ and Cu(II) or Ni(II). If we now compare the absorption profiles of the L′ and C bands created after the metal ion addition, they are identical and centered on the same wavelength interval. After all these observations, we have no reasons to doubt that Ni(II) and Cu(II) are coordinated in the same way in the two systems and then that C can form hetero-dinuclear complexes. In Figures S3–S6, the titration curves of C and L′ together with Ni(II) and Cu(II) ions in the range from 1 to 2 metal ion equivalent are reported.

### 2.5. Biological Studies

#### 2.5.1. IC_50_ Evaluation of Compound C with and without Photoactivation in Cells

Since we confirmed the ability of C to be activated by light in solution, we decided to verify if this characteristic makes them potential photosensitizers in cells. We chose A549 cells as target since they represent a solid lung tumor that currently has no effective cure and, in principle, it is suitable for PACT treatments. In this context, we evaluated the cytotoxicity in A549 using the MTT assay and inserting different periods of light irradiation in the protocol. Briefly, the MTT test consists of a 24 h treatment in which cells are exposed to the drug. During the treatment we expose cells for 3 h to light irradiation and in the end, we measured the cell viability using MTT. We limited the exposure to 3 h because we verified that cells suffered after this period of irradiation. In addition, to be sure to avoid the potential harmful effect of light we used, as control, cells that were not treated with the drug but were irradiated. The results of this experiment are reported in Table 1. Very good results were obtained for C that showed an IC_50_ of 84.62 μM in dark conditions and of 10.52 μM after 3 h of irradiation. We also tested the [(η^6^-p-cym)Ru(N,N-bipy)(DMSO)] complex and the pure free pro-ligand L for their IC_50_ values, and the results were >100 µM and 20.11 ± 0.27 µM, respectively.

#### 2.5.2. IC_50_ Evaluation of Compound C Treated with Copper(II) and Nickel(II) Chlorides

**Figure 5:**
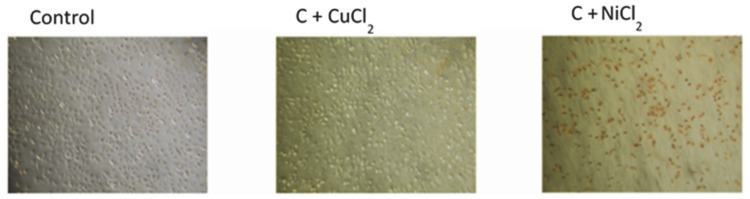
Pictures of A549 cells untreated and treated with C mixed with copper(II) and nickel(II) chlorides.

To test if C can increase the uptake of copper and nickel chlorides in cells through the formation of the above mentioned hetero-dinuclear complexes, we decided to compare the IC_50_ values of C, NiCl_2_, CuCl_2_ with those obtained from the administration of a 1:1 mixture of C with NiCl_2_ and CuCl_2_, respectively. The results are reported in Table 2 and show a strong increase in the cytotoxicity passing from the free components to the mixtures. However, the IC_50_ values are affected by uncertainty because the mixtures colored the medium and this affected the colorimetric quantification of MTT. To remedy to this setback, we show the pictures of cells treated with the two mixtures (Figure 5). They clearly show that cells suffered from the treatment with mixtures because they changed morphology, becoming more spherical and less attached to the well bottom: an unequivocal sign of cell damage. In addition, in cells treated with the mixture C + NiCl_2_, a change in the color of cells that clearly indicated the internalization of the compound was also visible.

## 3. Materials and Methods

### 3.1. Chemistry

All reactants used were purchased from Sigma Aldrich (Milan, Italy). The ^1^H NMR and ^13^C NMR spectra were recorded on a Bruker Anova spectrometer (Billerica, MA, USA) at 400 MHz. The FT-IR measurements were recorded on a Nicolet 5PC FTIR (Rodano, MI, Italy) analyzing products directly on the ATR accessory in the 4000–400 cm^−1^ range. The relative intensity of reported FT-IR signals is defined as s = strong, m = medium, and w = weak. ESI-MS analysis was performed using a Waters Acquity Ultraperformance ESI-MS spectrometer with Single Quadrupole Detector (Sesto San Giovanni, MI, Italy). Elemental analyses were performed on a CHNS Thermo Fischer (Rodano, MI, Italy). UV–vis spectra were recorded on a Thermo Scientific Evolution 260 Bio instrument (Rodano, MI, Italy). HR-MS were recorded on a Thermo Scientific Orbitrap LTQ-XL (Rodano, MI, Italy). The light intensity measurement was carried out with an Optical Power Meter (Newport 840-C, Monza, Italy) equipped with a Mod. 818-UV detector (active area 1 cm^2^). The determination was at 420 nm.

#### 3.1.1. Quinoline-2-carboxaldehydemonothiocarbohydrazone (L′)

**Figure 6:**
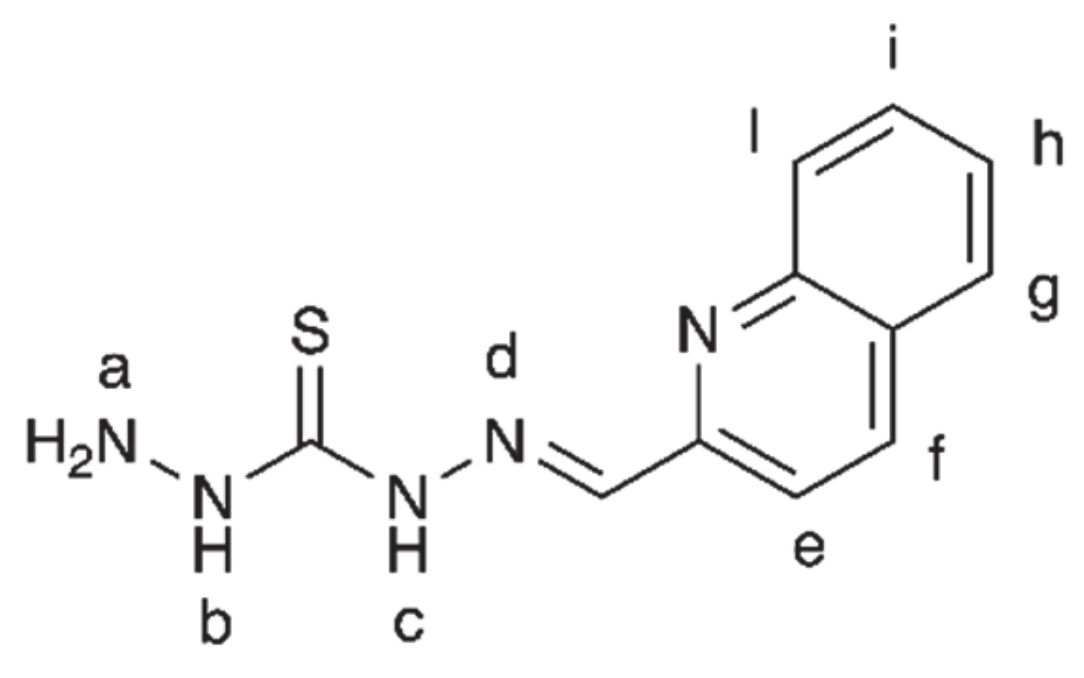
Quinoline-2-carboxaldehydemonothiocarbohydrazone (L′).

Thiocarbohydrazide (0.20 g, 1.88 mmol) was dissolved in 50 mL of methanol at 50 °C. At the same time, the quinoline-2-carboxaldehyde (0.30 g, 1.88 mmol) was dissolved in 5 mL of methanol and it was dripped slowly into the thiocarbohydrazide solution. The mixture was refluxed and stirred for 5 h. The product precipitated during the reaction. The solution was cooled to room temperature and left at −4 °C for 24 h. The product was collected by filtration, washed with hexane and dried under vacuum. Pale orange powder. Yield: 72%. FT-IR (cm^−1^): 1597 (s), 1095 (m), 829 (s). ^1^H-NMR (δ, ppm, DMSO-D_6_): 4.97 (s, 2H, H_a_), 7.62 (t, J = 7.4 Hz, 1H, H_l_), 7.77 (t, J = 7.7 Hz, 1H, H_i_), 7.99 (t, J = 8.8 Hz, 2H, H_g_, H_h_), 8.19 (s, 1H, H_d_), 8.36 (d, J = 8.7 Hz, 1H, H_f_), 8.55 (d, J = 8.7 Hz, 1H, H_e_), 10.20 (s, 1H, H_b_), 11.80 (s, 1H, H_c_) ^13^C-NMR (δ, ppm, 100 MHz, DMSO-D_6_): 118.81 (C-H quinoline), 127.55 (C-H quinoline), 128.26 (C quaternary quinoline), 128.38 (C-H quinoline), 129.21 (C-H quinoline), 130.37 (C-H quinoline), 136.68 (C-H quinoline), 142.78 (C-H imine), 147.77 (C quaternary quinoline), 154.50 (C quaternary quinoline), 176.55 (C quaternary thiocarbohydrazone) ESI-MS (+) *m*/*z* calc. 246.30 found 246.42. The structure of quinoline-2-carboxaldehydemonothiocarbohydrazone (L′) is shown is the Figure 6.

#### 3.1.2. Quinoline-2-carboxaldehyde-pyridine-4-formyl-bisthiocarbohydrazone (L)

**Figure 7:**
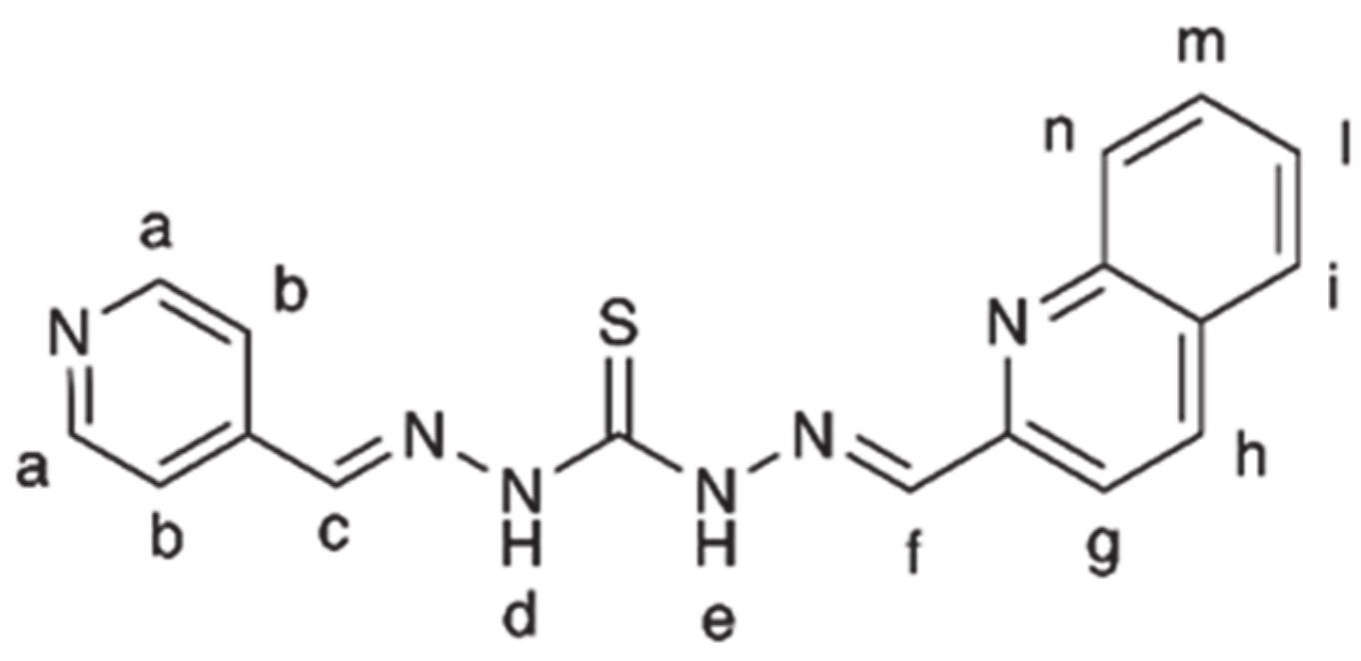
Quinoline-2-carboxaldehyde-pyridine-4-formyl-bisthiocarbohydrazone (L).

L′ (0.10 g, 0.401 mmol) was dissolved in 10 mL of THF at room temperature. After the dissolution of L′, 4-pyridinecarboxaldehyde (0.0429 g, 0.401 mmol) was added. The mixture was stirred for 0.5 h, then the solvent was removed under reduced pressure. Yield: 69%. ^1^H-NMR (δ, ppm, DMSO-D_6_): 7.65 (t, J = 8 Hz, 2H, H_m_, H_n_), 7.81 (t, J = 7.6 Hz, 2H, H_b_), 8.04 (t, J = 8.8 Hz, 3H, H_i_, H_l_, H_f_), 8.46 (d, J = 8.4 Hz, 2H, H_h_, H_c_), 8.69 (d, J = 5.3 Hz, 3H, H_a_, H_g_), 12.06 (bs, 1H, H_e_), 12.45 (bs, 1H, H_d_) ^13^C-NMR (δ, ppm, 100 MHz, DMSO-D_6_): 118.76 (C-H quinoline), 121.89 (C-H pyridine), 127.51 (C-H quinoline), 128.39 (C-H quinoline), 128.68 (C quaternary quinoline), 129.58 (C-H quinoline), 130.53 (C-H quinoline), 137.16 (C-H quinoline), 138.84 (C-H imine), 141.42 (C quaternary pyridine), 142.83 (C-H imine), 147.86 (C quaternary quinoline), 150.56 (C-H pyridine close to N), 150.65 (C quaternary quinoline), 176.06 (C quaternary thiocarbohydrazone) ESI-MS (+) *m*/*z* calc. 334.40 found 334.15. The structure of quinoline-2-carboxaldehyde-pyridine-4-formyl-bisthiocarbohydrazone (L) is shown is the Figure 7.

#### 3.1.3. Dichloro(η^6^-p-cymene)ruthenium(II) Dimer

**Figure 8:**
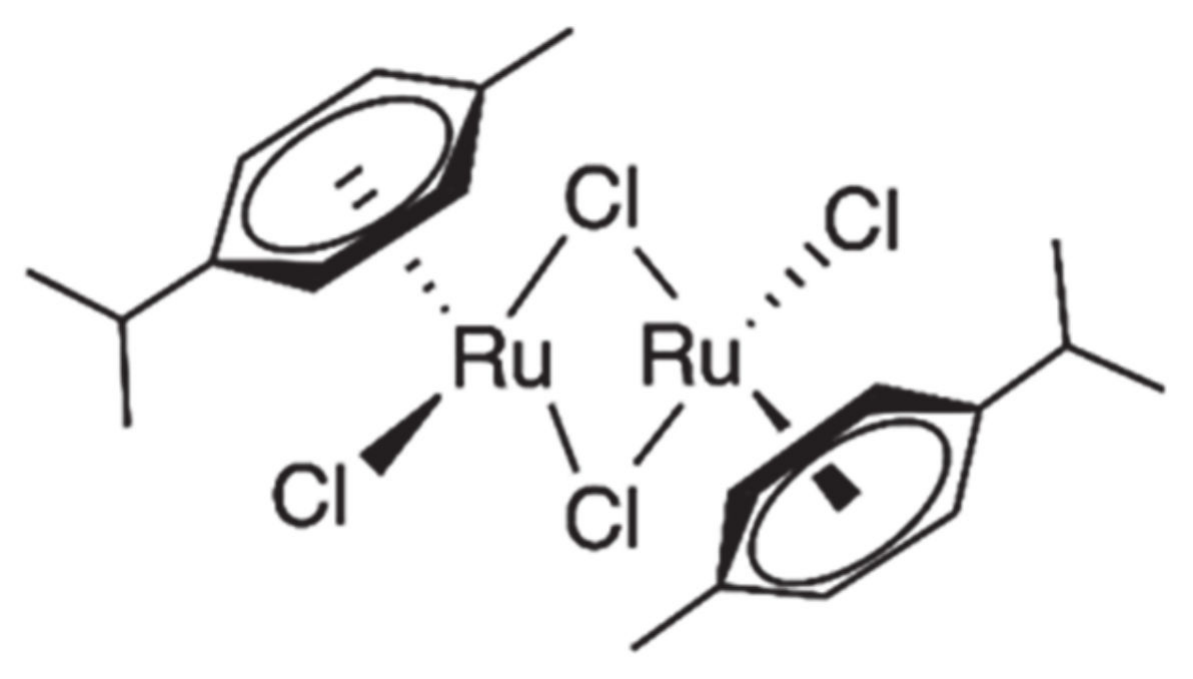
Dichloro(η^6^-p-cymene)ruthenium(II) dimer.

An amount of 1.0 g of RuCl_3_∙nH2O was dissolved in 10 mL of methanol, then a large excess of α-phellandrene (3 mL) was added. The reaction was performed using a microwave reactor at 150 W and 140 °C for 10 min. At the end, 10 mL of pentane was added to precipitate the product, which was collected by filtration, washed with pentane (3 × 5 mL), and dried under vacuum. Dark red crystals. Yield: Quantitative ^1^H-NMR (δ, ppm, 400 MHz, CDCl_3_): 1.30 (d, J = 6.9 Hz, 6H), 2.18 (s, 3H), 2.94 (hept, 1H), 5.35 (d, J = 6.0 Hz, 2H), 5.49 (d, J = 6.0 Hz, 2H). ^13^C-NMR (δ, ppm, 100 MHz, CDCl_3_): 101.23 (C quaternary p-cymene), 96.76 (C quaternary p-cymene), 81.31 (C-H aromatic p-cymene), 80.54 (C-H aromatic p-cymene), 30.64 (CH_3_ p-cymene), 22.17 (CH_3_ isopropyl p-cymene), 18.95 (CH isopropyl p-cymene). The structure of dichloro(η^6^-p-cymene)ruthenium(II) dimer is shown is the Figure 8.

#### 3.1.4. [(η6-p-cym)Ru(4-formylpyridine)Cl_2_] (IC1)

**Figure 9:**
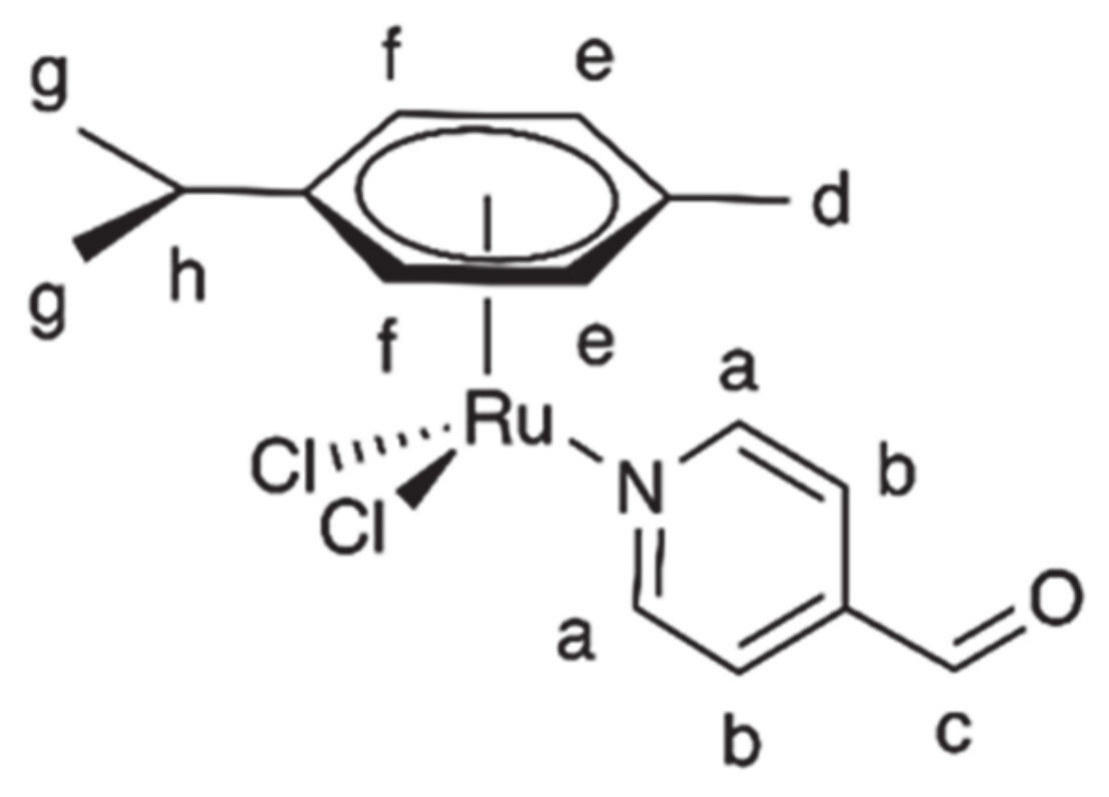
[(η^6^-p-cym)Ru(4-formylpyridine)Cl_2_] (IC1).

[(η^6^-p-cym)RuCl_2_]_2_ (0.10 g, 0.16 mmol) and 4-formylpyridine (0.03 g, 0.32 mmol) were dissolved in 15 mL of dry CH_2_Cl_2_ in a Schlenk flask with a nitrogen atmosphere. The mixture was stirred for 5 h, then 10 mL of diethyl ether was added, and the solution was left al −4 °C overnight. The precipitate was collected by filtration, washed with cold diethyl ether (2 × 5 mL), and dried under vacuum. Orange solid. Yield: 90%. ^1^H NMR (δ, ppm, 400 MHz, CDCl_3_): 1.34 (d, J = 6.9 Hz, 6H, H_g_), 2.14 (s, 3H, H_d_), 3.02 (hept, J = 6.9 Hz, 1H, H_h_), 5.28 (d, J = 6.0 Hz, 2H, H_f_), 5.50 (d, J = 6.0 Hz, 2H, H_e_), 7.73 (d, J = 6.5 Hz, 2H, H_b_), 9.37 (d, J = 6.5 Hz, 2H, H_a_), 10.13 (s, 1H, H_c_) (Figure S9) ^13^C-NMR (δ, ppm, 100 MHz, CDCl_3_): 18.29 (CH isopropyl p-cymene), 22.30 (CH_3_ isopropyl p-cymene), 30.73 (CH_3_ p-cymene), 82.44 (C-H aromatic p-cymene), 83.08 (C-H aromatic p-cymene), 97.50 (C quaternary p-cymene), 104.00 (C quaternary p-cymene), 122.58 (C-H pyridine close to aldehyde), 141.91 (C quaternary pyridine), 156.61 (C-H pyridine close to N), 189.96 (C quaternary aldehyde) (Figure S10) ESI-MS (+) [M-Cl^−^]^+^ *m*/*z* calc. 377.85 found 377.51. HR-MS: [M-Cl + MeOH]^+^ calculated: 410.04607; found: 410.04553 (Figure S17). CHNS analysis: C_16_H_19_Cl_2_NORu: C: 46.50, H: 4.63, N: 3.39; found: C: 46.63, H: 4.50, N: 3.34. The structure of [(η^6^-p-cym)Ru(4-formylpyridine)Cl_2_] (IC1) is shown is the Figure 9.

#### 3.1.5. [(η6-p-cym)Ru(N,N′-bpy)(4-formylpyridine)](CF_3_ SO_3_)_2_ (IC2)

**Figure 10:**
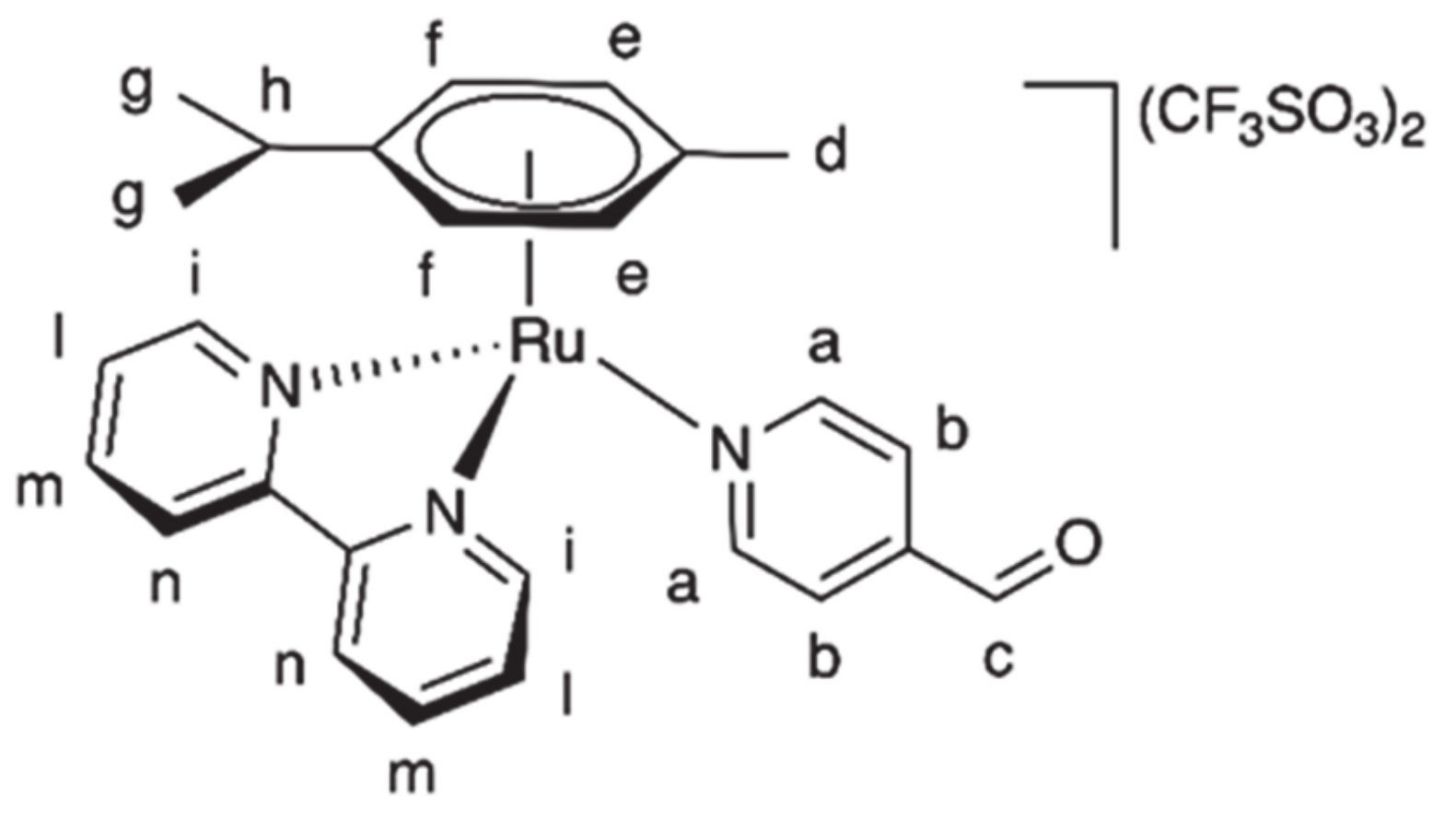
[(η^6^-p-cym)Ru(N,N′-bpy)(4-formylpyridine)]( CF_3_ SO_3_)_2_ (IC2).

IC1 (0.10 g, 0.22 mmol) was dissolved in 15 mL of dry CH_2_Cl_2_ in a Schlenk flask with a nitrogen atmosphere. At the same time, an AgCF_3_SO_3_ (0.11 g, 0.43 mmol) solution was prepared in 3 mL of dry methanol in a nitrogen atmosphere, and then it was dropped slowly into the ruthenium complex solution. The solution became turbid immediately and it was stirred for 2 h at room temperature, covering the flask with aluminum foil to keep the solution in dark conditions. Then, the AgCl formed was removed filtering the solution in a Schlenk filter and the clear orange solution obtained was dropped in a Schlenk flask with bipyridine (0.03 g, 0.22 mmol). The mixture was stirred overnight always in a nitrogen and dark environment. In the end, the solvent was removed under reduced pressure and the orange solid obtained was washed with diethyl ether (3 × 10 mL) and dried under vacuum. Orange powder. Yield: 53%. ^1^H-NMR (δ, ppm, 400 MHz, CD_6_CO): 0.99 (d, J = 6.9 Hz, 6H, H_g_), 1.97 (s, 3H, H_d_), 2.67 (hept, J = 6.9 Hz, 1H, H_h_), 6.47 (d, J = 6.3 Hz, 2H, H_f_), 6.86 (d, J = 6.3 Hz, 2H, H_e_), 7.90 (d, J = 6.2 Hz, 2H, H_n_), 8.08 (m, 2H, H_m_), 8.47 (t, J = 7.9 Hz, 2H, H_l_), 8.68 (d, J = 8.0 Hz, 2H, H_b_), 9.11 (d, J = 6.3 Hz, 2H, H_a_), 10.13 (s, 1H, H_c_), 10.19 (d, J = 5.8 Hz, 2H, H_i_) (Figure S11) ^13^C-NMR (δ, ppm, 100 MHz, CD_6_CO): 17.22 (CH isopropyl p-cymene), 21.52 (CH_3_ isopropyl p-cymene), 30.77 (CH_3_ p-cymene), 84.96 (C-H aromatic p-cymene), 92.64 (C-H aromatic p-cymene), 104.50 (C quaternary p-cymene), 109.37 (C quaternary p-cymene), 124.76 (C-H pyridine close to aldehyde), 124.98 (C-H bipyridine), 129.58 (C-H bipyridine), 141.40 (C-H bipyridine), 143.65 (C quaternary pyridine), 155.17 (C-H bipyridine close to N), 155.55 (C quaternary bipyridine), 156.90 (C-H pyridine close to N), 190.65 (C quaternary aldehyde) (Figure S12) ESI-MS (+) M-SO_3_CF_3_]^+^ *m*/*z* calc. 647.65 found. 680.11 (hemiacetal with methanol). HR-MS: [M-2CF_3_SO_3_-C_6_H_5_NO]^2+^calculated: 196.04132; found: 196.04094. [M+MeOH]^2+^; calculated: 265.57230; found: 265.57244. [M-SO_3_CF_3_]^+^; calculated: 648.07178; found: 648.07124 (Figure S18). CHNS analysis: C_28_H_27_F_6_N_3_O_7_RuS_2_: calculated: C: 42.21, H: 3.42, N: 5.27, S: 8.05; found: C: 42.01, H: 3.14, N: 5.32, S: 8.38. The structure of [(η^6^-p-cym)Ru(N,N′-bpy)(4-formylpyridine)](CF_3_ SO_3_)_2_ (IC2) is shown is the Figure 10.

#### 3.1.6. [(η6-p-cym)Ru(N,N′-bpy)Cl](PF_6_) (IC3)

**Figure 11:**
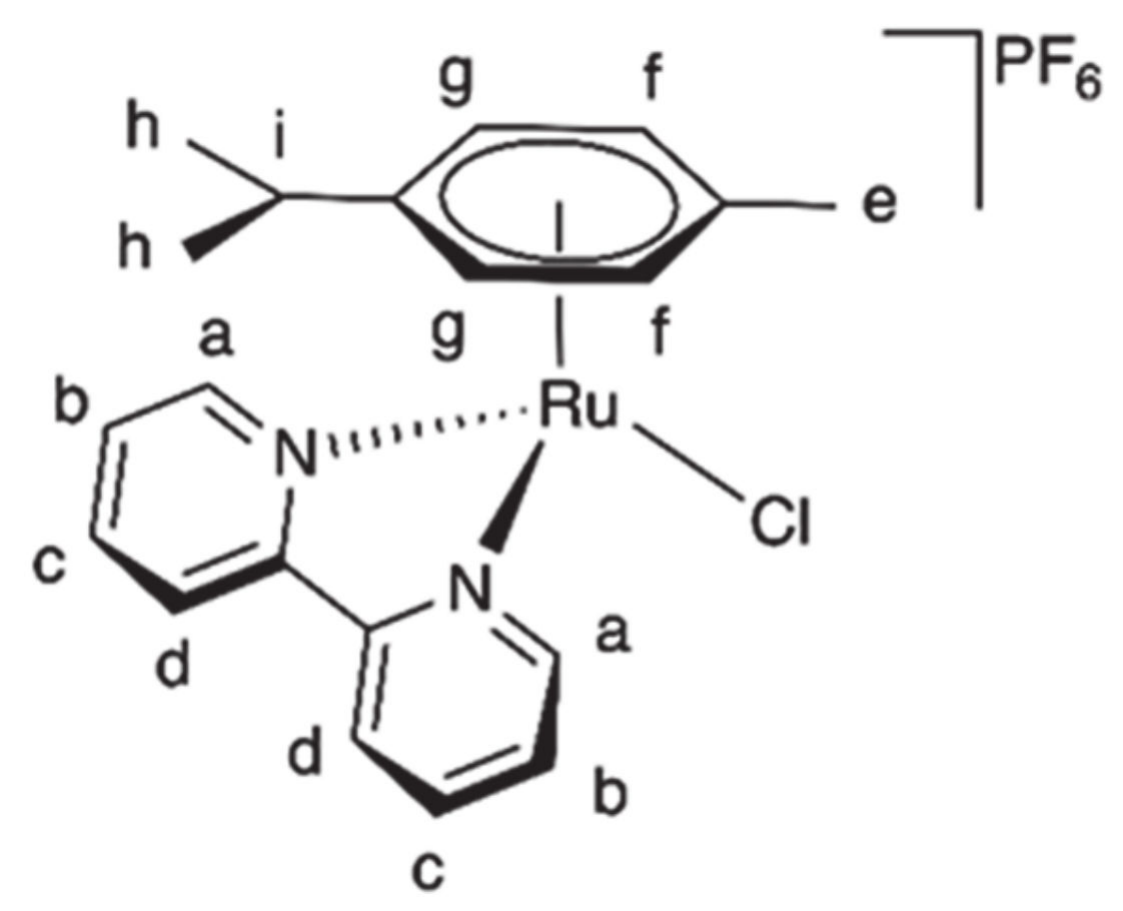
[(η6-p-cym)Ru(N,N′-bpy)Cl](PF_6_) (IC3).

[(η^6^-p-Cym)RuCl_2_]_2_ (0.150 g, 0.25 mmol, 1 eq.), 2,2′-bipyridine (0.076 g, 0.49 mmol, 2 eq.) and KPF_6_ (0.090 g, 0.49 mmol, 2 eq.) were dissolved in dry methanol in a Schlenk flask with a nitrogen atmosphere. The mixture was stirred for 4 h, and during the reaction KCl was formed like a white precipitate. The KCl was separated with filtration; then, diethyl ether was added, and the solution was left al −4 °C overnight. The precipitate was collected by filtration, washed with cold diethyl ether and dried under vacuum. Yellow solid. Yield: 71% ^1^H-NMR (δ, ppm, 400 MHz, CD_6_CO): 1.12 (d, J = 7.2 Hz, 6H, H_h_), 2.33 (s, 3H, H_e_), 2.80 (m, 1H, H_i_), 6.02 (d, J = 6.0, 2H, H_g_), 6.27 (d, J = 6.0, 2H, H_f_), 7.85 (m, 2H, H_c_), 8.36 (m, 2H, H_b_), 8.65 (d, J = 8.4, 2H, H_d_), 9.63 (d, J = 5.6, 2H, H_a_) ^1^H-NMR (δ, ppm, 400 MHz, DMSO-D_6_): 0.94 (d, J = 7.2 Hz, 6H, H_h_), 2.19 (s, 3H, H_e_), 2.58 (m, 1H, H_i_), 5.99 (d, J = 6.0, 2H, H_g_), 6.24 (d, J = 6.0, 2H, H_f_), 7.80 (t, J = 6.4 Hz, 2H, H_c_), 8.29 (t, J = 6.4 Hz, 2H, H_b_), 8.66 (d, J = 8.4, 2H, H_d_), 9.56 (d, J = 5.6, 2H, H_a_) (Figure S13) ^13^C-NMR (δ, ppm, 100 MHz, CD_6_CO): 17.96 (C-H isopropyl p-cymene), 21.35 (CH_3_ isopropyl p-cymene), 30.96 (CH_3_ p-cymene), 84.49 (C-H p-cymene), 86.75 (C-H p-cymene), 103.88 (C quaternary p-cymene), 105.10 (C quaternary p-cymene), 123.75 (C-H bipyridine), 127.65 (C-H bipyridine), 139.91 (C-H bipyridine), 154.91 (C quaternary bipyridine), 155.76 (C-H bipyridine close to N) (Figure S14) ESI-MS (+) *m*/*z* [M-CH_3_PF_6_]^+^ calc. 427.05 found 427.17 CHNS analysis: C_20_H_22_N_2_PF_6_ClRu: calculated: C: 42.00, H: 3.88, N: 4.90. found: C: 41.73, H: 3.94, N: 4.84. The structure of [(η6-p-cym)Ru(N,N′-bpy)Cl](PF_6_) (IC3) is shown is the Figure 11.

#### 3.1.7. [(η^6^-p-cym)Ru(N,N′-bpy)(N-4-formylpyridine-2-quinolinecarboxaldehydethiocarbohydrazone)](SO_3_CF_3_)_2_ (C)

**Figure 12:**
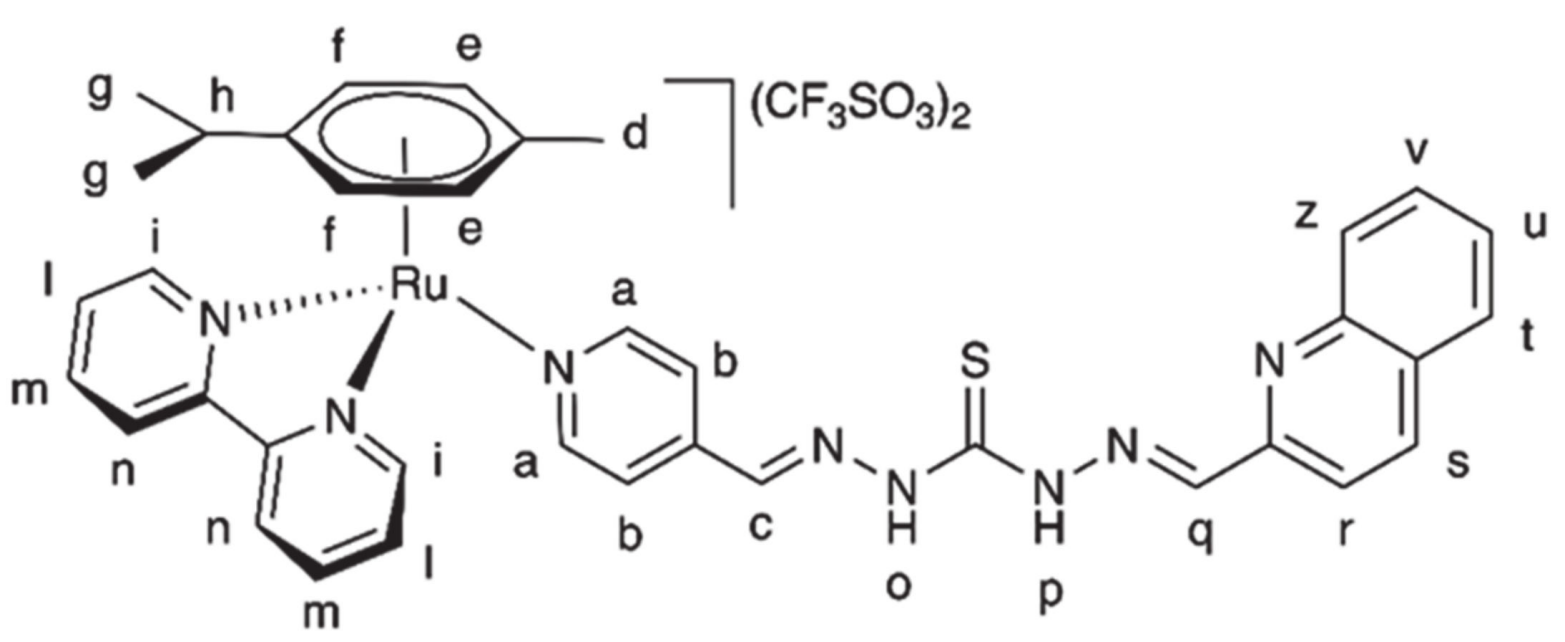
[(η^6^-p-cym)Ru(N,N′-bpy)(N-4-formylpyridine-2-quinolinecarboxaldehydethiocarbohydrazone)](SO_3_CF_3_)_2_ (C).

IC2 (0.10 g, 0.13 mmol) and L′ (0.03 g, 0.13 mmol) were dissolved in 20 mL of tetrahydrofuran dry in a Schlenk flask in nitrogen atmosphere. The mixture was stirred for 24 h at room temperature covering the flask with aluminum foil to keep the solution in the dark. The mixture was then microfiltered on celite and the solvent of the clear solution obtained was removed under reduced pressure. Finally, the solid obtained was washed three times with diethyl ether and dried under vacuum. Orange solid. Yield: 82%. ^1^H-NMR (δ, ppm, 400 MHz, CD_6_CO): 0.84 (d, J = 6.9 Hz, 6H, H_g_), 1.82 (s, 3H, H_d_), 2.48 (m, 1H, H_h_), 6.26 (d, J = 6.4 Hz, 2H, H_f_), 6.69 (d, J = 6.4 Hz, 2H, H_e_), 7.66 (m, 1H, H_s_), 7.82 (m, 2H, H_n_), 7.91 (m, 1H, H_v_), 8.01 (m, 1H, H_u_), 8.08 (m, 3H, H_m_, H_t_), 8.46 (m, 5H, H_l_, H_b_, H_q_), 8.68 (m, 3H, H_c_, H_r_, H_z_), 8.76 (d, J = 6.7 Hz, 2H, H_a_), 10.18 (m, 2H, H_i_), 12.21 (bs, 1H, H_o_), 12.58 (bs, 1H, H_p_) ^1^H-NMR (δ, ppm, 400 MHz, DMSO-D_6_): 0.84 (d, J = 6.9 Hz, 6H, H_g_), 1.82 (s, 3H, H_d_), 2.46 (m, 1H, H_h_), 6.26 (d, J = 6.3 Hz, 2H, H_f_), 6.68 (d, J = 6.3 Hz, 2H, H_e_), 7.65 (t, J = 8 Hz, 2H, H_z_, H_v_), 7.81 (t, J = 7.6 Hz, 2H, H_n_), 8.04 (m, 6H, H_t_, H_u_, H_l_, H_m_), 8.43 (m, 6H, H_s_, H_c_, H_b_, H_r_, H_q_), 8.63 (m, 2H, H_a_), 9.89 (m, 2H, H_i_), 12.19 (bs, 1H, H_o_), 12.53 (bs, 1H, H_p_) (Figure S15) ^13^C-NMR (δ, ppm, 100 MHz, CD_6_CO): 17.28 (CH isopropyl p-cymene), 21.53 (CH_3_ isopropyl p-cymene), 30.85 (CH_3_ p-cymene), 85.01 (C-H aromatic p-cymene), 92.25 (C-H aromatic p-cymene), 104.70 (C quaternary p-cymene), 108.91 (C quaternary p-cymene), 119.81 (C-H quinoline), 118.83 (C-H quinoline), 122.97 (C-H imine), 123.89 (C quaternary pyridine), 124.69 (C-H bipyridine), 127.45 (C-H quinoline), 127.98 (C-H bipyridine), 128.38 (C quaternary quinoline), 129.05 (C-H quinoline), 129.53 (C-H quinoline), 129.98 (C-H quinoline), 136.59 (C quaternary quinoline), 138.13 (C-H imine), 141.33 (C-H bipyridine), 147.81 (C-H pyridine close to aldehyde),148.45 (C quaternary quinoline), 153.44 (C-H bipyridine close to N), 155.51 (C quaternary bipyridine), 156.78 (C-H pyridine close to N), 176.31 (C quaternary thiocarbohydrazone) (Figure S16) ESI-MS (+) [M-SO_3_CF_3_]^+^ *m*/*z* calc. 874.96 found 875.35 CHNS analysis: C_39_H_36_N_8_S_3_RuF_6_: calculated: C: 45.74, H: 3.54, N: 10.94, S: 9.39 found: C: 45.99, H: 3.83, N: 11.05, S: 9.76. The structure of [(η^6^-p-cym)Ru(N,N′-bpy)(N-4-formylpyridine-2-quinolinecarboxaldehydethiocarbohydrazone)](SO_3_CF_3_)_2_ (C) is shown is the Figure 12.

### 3.2. Biology

#### 3.2.1. Cell Lines

The A549 cells were purchased from the American Type Culture Collection (ATCC, Manassas, VA, USA). Cells were grown in RPMI 1640 medium containing 10% heat-inactivated fetal bovine serum, 100 U mL^−1^ penicillin and streptomycin at 37 °C with 5% CO_2_. Experiments were conducted with cells in the log phase and A549 adherent cells were harvested using trypsin.

#### 3.2.2. Morphological Observations

To observe the cell morphology, A549 cells were cultured and treated with compounds on glass slides. After 24 h exposure, the medium was removed, the specimens were fixed, stained with hematoxylin and eosin, and observed through a light microscope Olympus (Olympus, Tokyo, Japan).

#### 3.2.3. Cell Viability Assay

Drug effects on cell viability were analyzed using a 3-(4,5-dimethylthiazol-2-yl)-2,5-diphenyltetrazolium bromide (MTT) colorimetric assay, based upon the ability of metabolically active cells to reduce MTT into formazan by the action of mitochondrial dehydrogenases. A549 (70,000 mL^−1^) cells were seeded into 96-well plates overnight, and then exposed to compounds at indicated concentrations. At the end of the treatment, MTT was added (final concentration 0.5 mg mL^−1^) for 3 h at 37 °C and formazan crystals were dissolved in 100 mL for each well of acidic isopropanol (0.08 N HCl). After mixing, absorbance was evaluated using a Multiskan Ascent microwell plate reader equipped with a 550 nm filter (Thermo Labsystems, Helsinki, Finland). At least three independent experiments were performed with eight replicate wells per sample. The half maximal inhibitory concentration (IC_50_) was determined as the concentration resulting in 50% cell growth reduction compared with untreated control cells.

## 4. Conclusions

A Ru(II) complex with an asymmetrical thiocarbohydrazone acting as a monodentate ligand was synthesized in good yields. The Ru(II) complex obtained is stable under physiological conditions. Photoactivation studies highlighted the ability of the complex to dissociate upon irradiation. In addition, this Ru(II) complex is able, thanks to its quinoline moiety, to bind metal ions such as Cu(II) and Ni(II) giving rise to heterobimetallic complexes. The Ru(II) complex shows an increased ability to inhibit A549 tumor cell proliferation when irradiated, proving to be a good candidate for PACT. The heterobimetallic complexes are more active than the Ru(II) complex alone in the dark, and morphological studies demonstrate the cell internalization of Ru(II)/Ni(II) bimetallic complex.

## Figures and Tables

**Table 1:** Half maximal inhibitory concentration (IC_50_) values of A549 cells treated with Ru(II) half-sandwich complex C in dark conditions and after 3 h of irradiation.

	IC_50_ (μM) A549 24 h	
	Dark	Upon 3 h of Irradiation
C	84.62 ± 3.90	10.52 ± 1.95

**Table 2:** IC_50_ values determined for A549 cells treated with C mixed with copper(II) and nickel(II) chlorides.

IC_50_ (μM) A549 24 h				
C	CuCl_2_	C + CuCl_2_	NiCl_2_	C + NiCl_2_
84.62 ± 3.90	>100	~20	>100	~10

## Data Availability

The data presented in this study are available in this article and in the Supplementary Informations file.

## Supplementary Materials

The following are available online, Figure S1. Comparison between the UV-vis spectra of the complex (20 µM) collected in PBS + 2% DMSO. Spectra were recorded immediately after the sample preparation (blue line) and after 24 h (red line), Figure S2. Photo-oven used for the photoactivation experiments, Figure S3. UV-titration of C with copper(II) chloride, Figure S4. UV-titration of C with nichel(II) chloride, Figure S5. UV-titration of L′ with copper(II) chloride, Figure S6. UV-titration of L′ with nichel(II) chloride, Figure S7. Comparison of the 1H-NMR spectrum of the complex C after 8 h of irradiation with the spectra of the pure [(η6-p-cym)Ru(N,N-bipy)(DMSO)] and the pure free pro-ligand, Figure S8. Suggested structure of the hetero-dinuclear complexes, Figure S9. 1H-NMR IC1 in CDCl3, Figure S10. 13C-NMR IC1 in CDCl3, Figure S11. 1H-NMR IC2 in CD6CO, Figure S12. 13C-NMR IC2 in CD6CO, Figure S13. 1H-NMR IC3 in DMSO-D6, Figure S14. 13C-NMR IC3 in CD6CO, Figure S15. 1H-NMR C in DMSO-D6, Figure S16. 13C-NMR C in CD6CO, Figure S17. Full HR-MS of IC1, Figure S18. Full HR-MS of IC2.
