# Supplementary material for: A New Photoactivatable Ruthenium(II) Complex with an Asymmetric Bis-Thiocarbohydrazone: Chemical and Biological Investigations
Source: Molecules. 2021 Feb 10;26(4):939. doi: 10.3390/molecules26040939 (PMC7916603; doi:10.3390/molecules26040939)
Supplement: Supplementary file 1 [file molecules-26-00939-s001.pdf]

molecules

MDPI

Supplementary Information

A New Photoactivatable Ruthenium(II) Complex with an Asymmetric Bis-Thiocarbohydrazone: Chemical and Biological Investigations

Marianna Pioli [1], Nicolò Orsoni [1], Mirco Scaccaglia [1], Rossella Alinovi [2,3], Silvana Pinelli [2,3], Giorgio Pelosi [1,3] and Franco Bisceglie [1,4,*]

1 Department of Chemistry, Life Sciences and Environmental Sustainability, University of Parma, 43124 Parma, Italy; pioli.marianna@gmail.com (M.P.); nicolo.orsoni@studenti.unipr.it (N.O.); mirco.scaccaglia@unipr.it (M.S.); giorgio.pelosi@unipr.it (G.P.)
2 Department of Medicine and Surgery, University of Parma, 43126 Parma, Italy; rossella.alinovi@unipr.it (R.A.); silvana.pinelli@unipr.it (S.P.)
3 C.I.R.C.M.S.B. Consorzio Interuniversitario di Ricerca in Chimica nei Sistemi Biologici, Parma Local Unit, 43124 Parma, Italy
4 C.O.M.T. Centre for Molecular and Translational Oncology, University of Parma, 43124 Parma, Italy
* Correspondence: franco.bisceglie@unipr.it

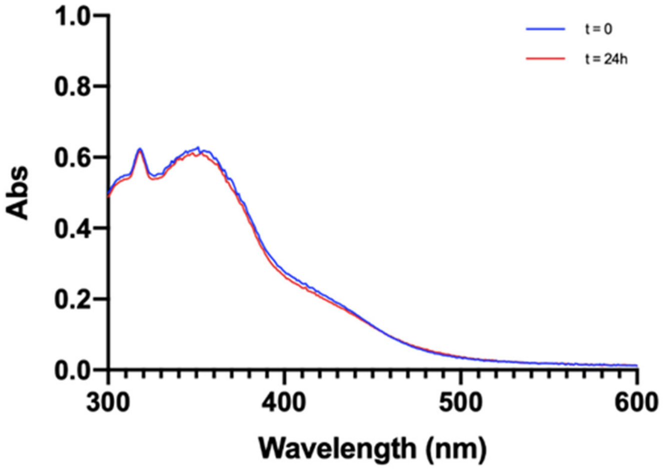

Figure S1. Comparison between the UV-vis spectra of the complex (20 μM) collected in PBS + 2% DMSO. Spectra were recorded immediately after the sample preparation (blue line) and after 24 h (red line).

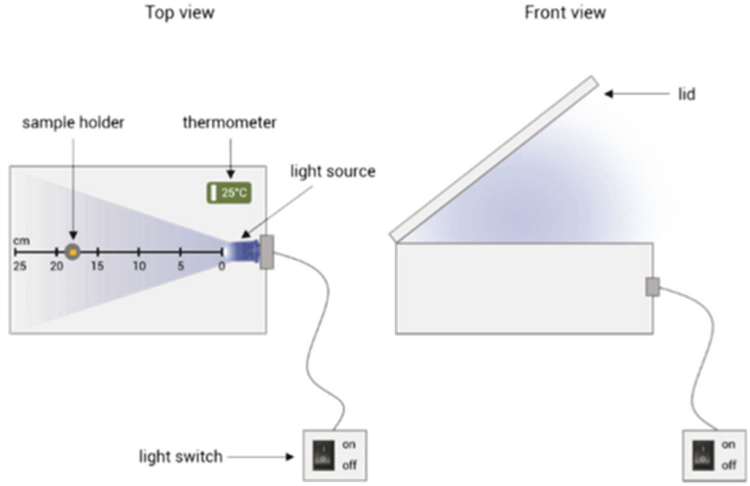

Figure S2. Photo-oven used for the photoactivation experiments.

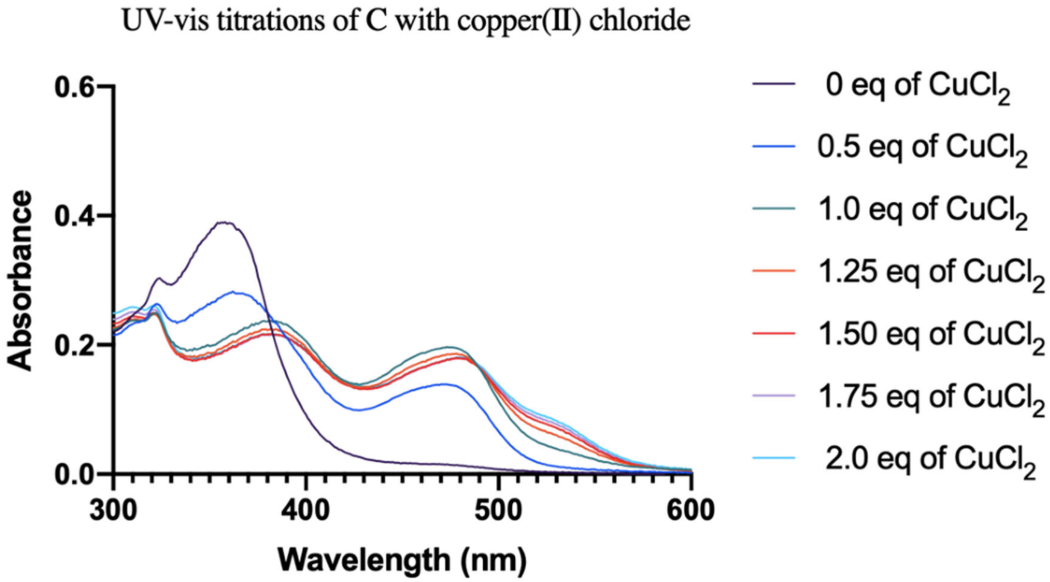

Figure S3. UV-titration of C with copper(II) chloride.

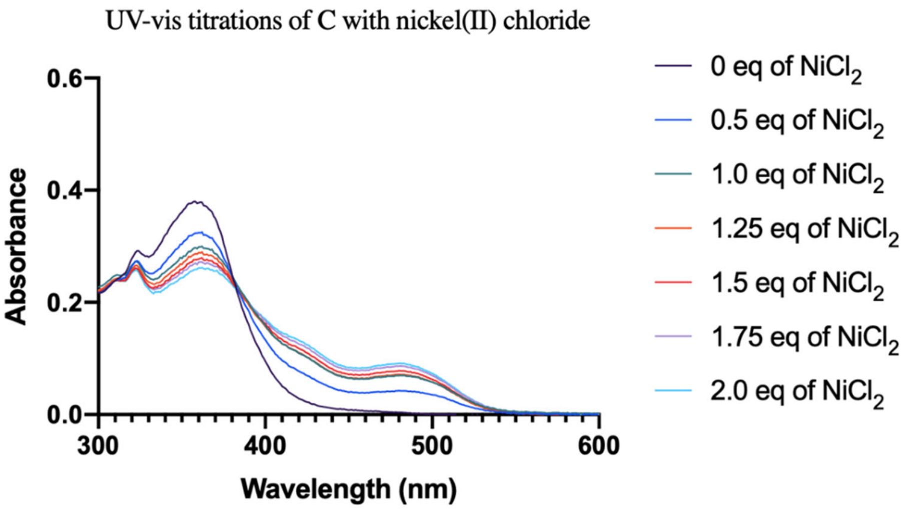

Figure S4. UV-titration of C with nickel(II) chloride.

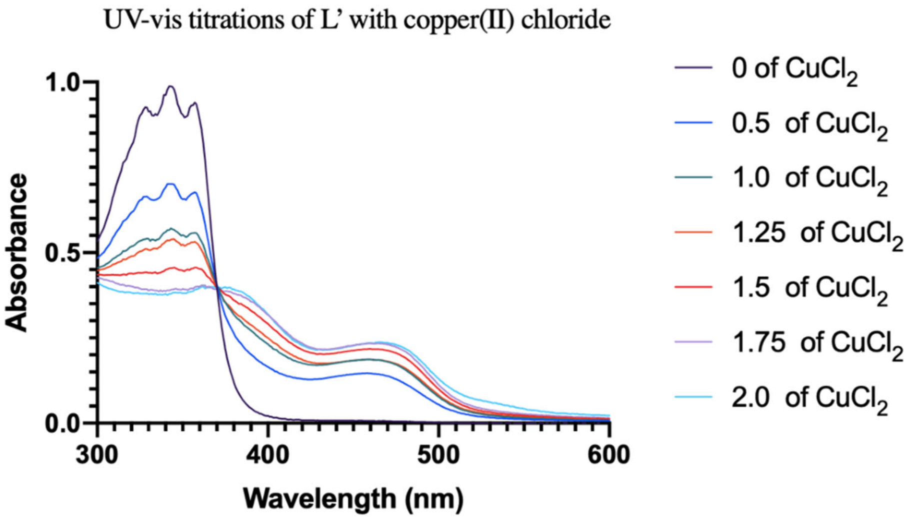

Figure S5. UV-titration of L′ with copper(II) chloride.

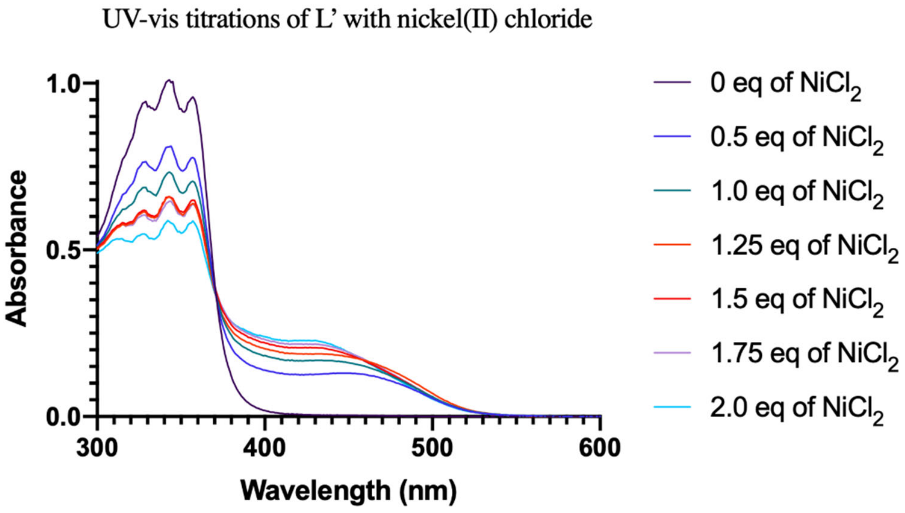

Figure S6. UV-titration of L′ with nickel(II) chloride.

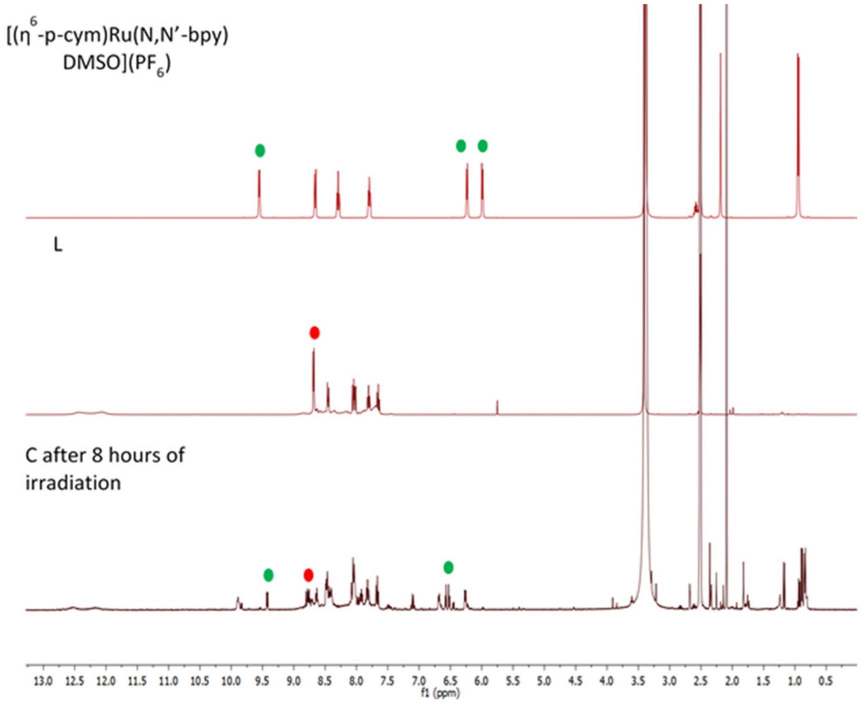

Figure S7. Comparison of the ^{1}H-NMR spectrum of the complex C after 8 h of irradiation with the spectra of the pure [(η^6-p-cym)Ru(N,N-bipy)(DMSO)] and the pure free pro-ligand.

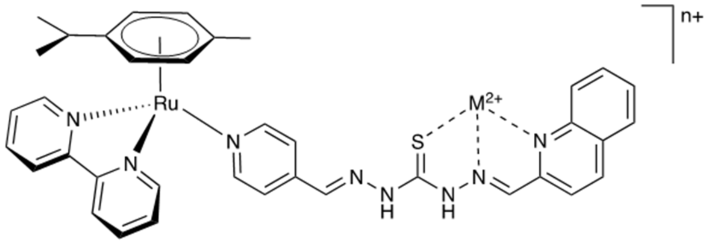

Figure S8. Suggested structure of the hetero-dinuclear complexes.

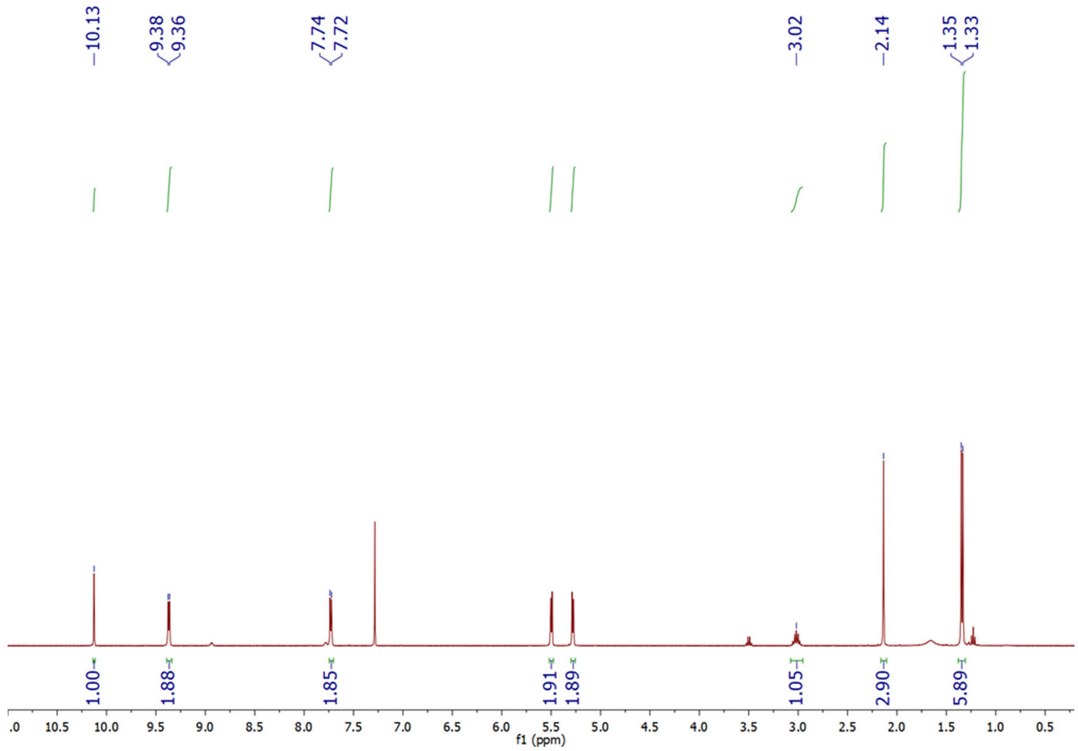

Figure S9. ^{1}H-NMR IC1 in $CDCl_3$.

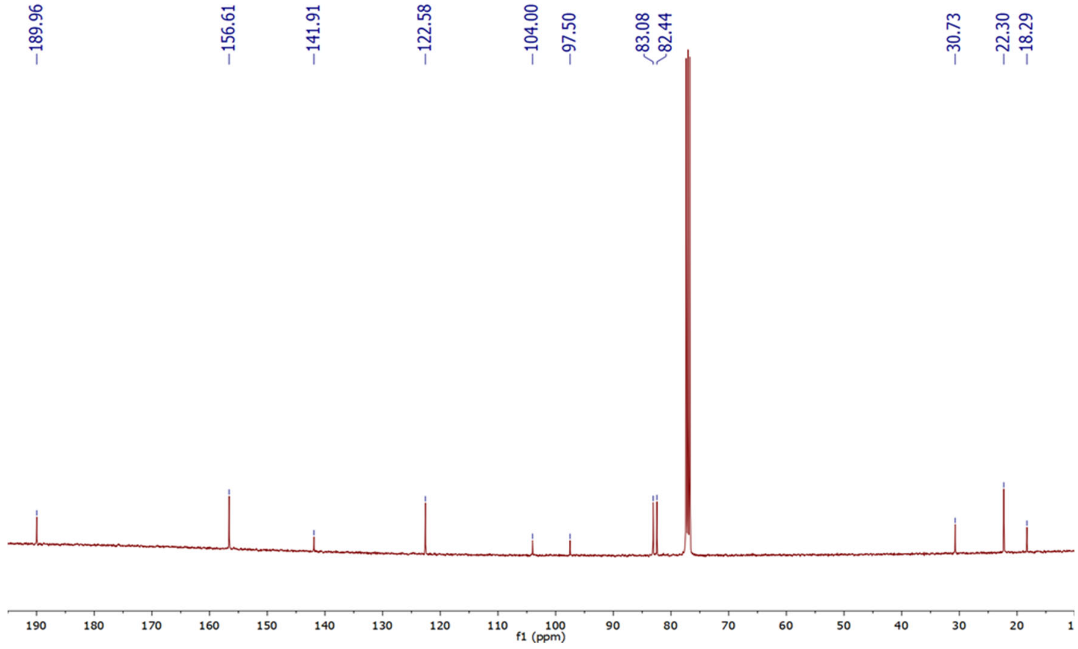

Figure S10. ^{13}C-NMR IC1 in $CDCl_3$.

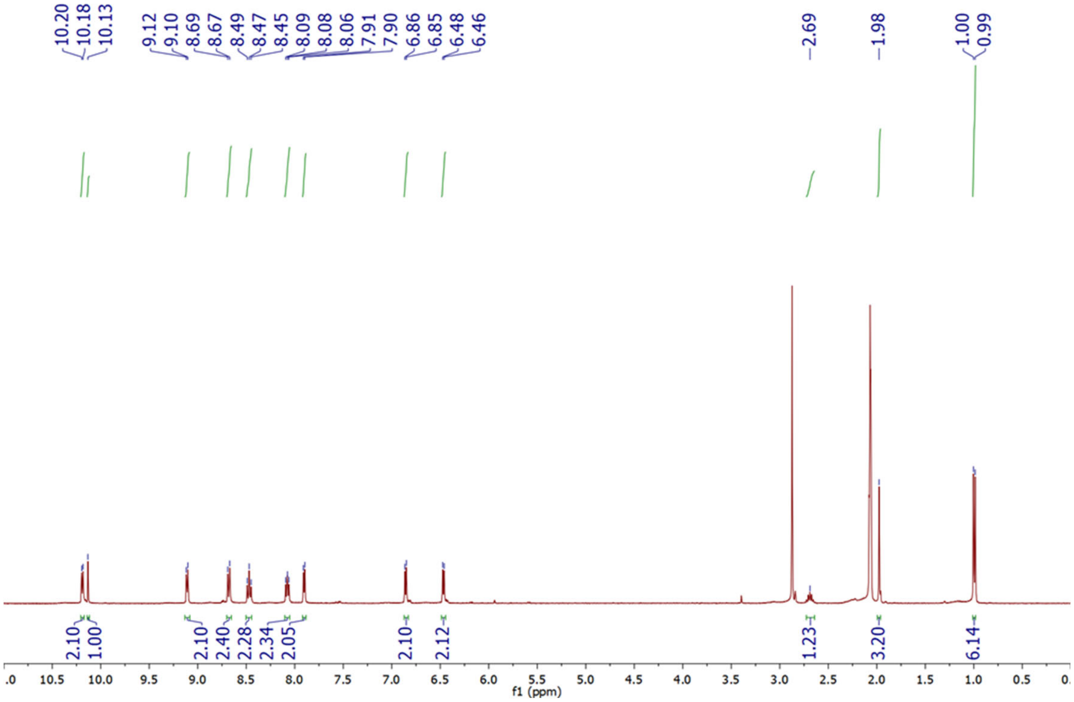

Figure S11. ^{1}H-NMR IC2 in CD_6CO.

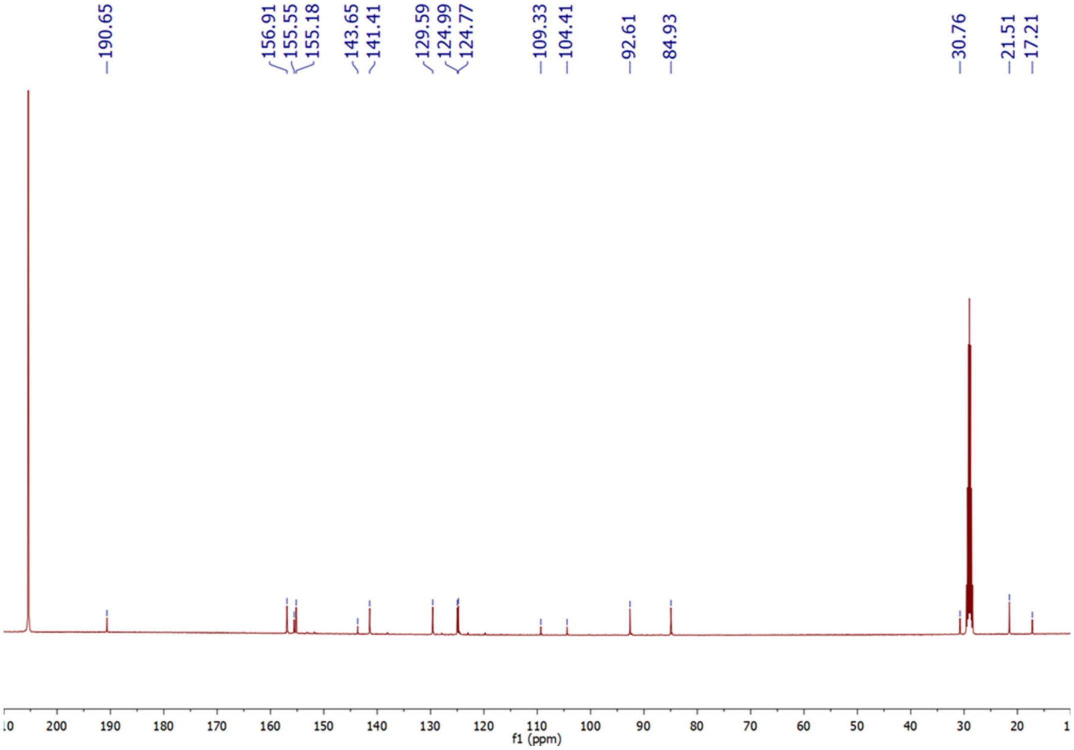

Figure S12. ^{13}C-NMR IC2 in CD_6CO.

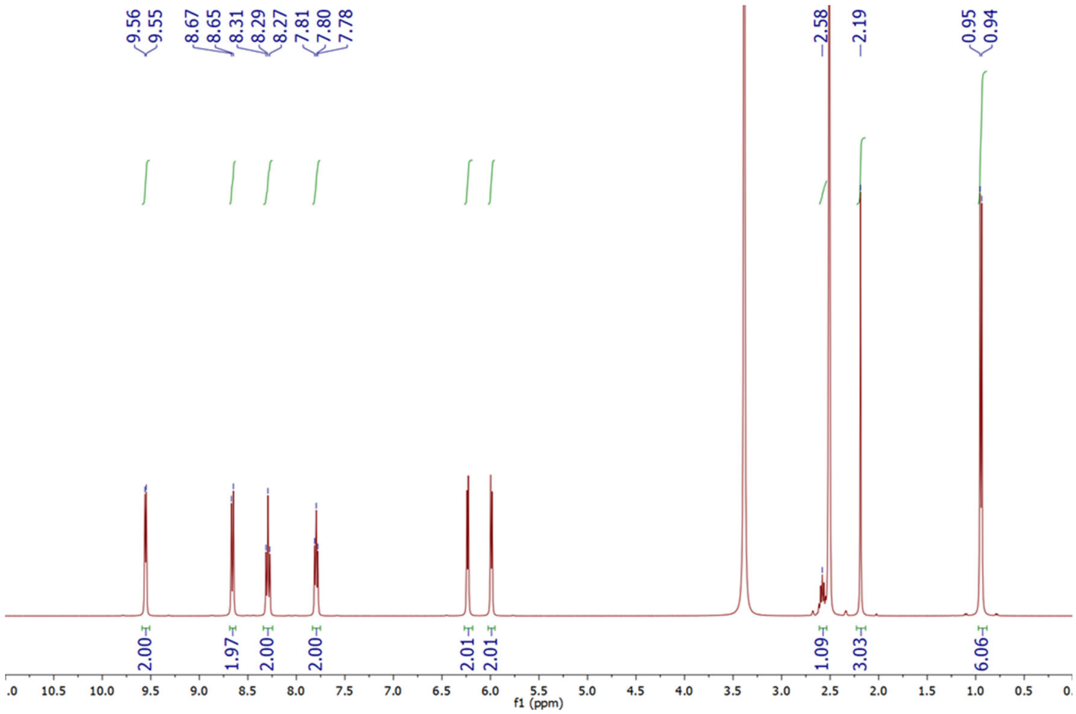

Figure S13. ^{1}H-NMR IC3 in DMSO-D_6.

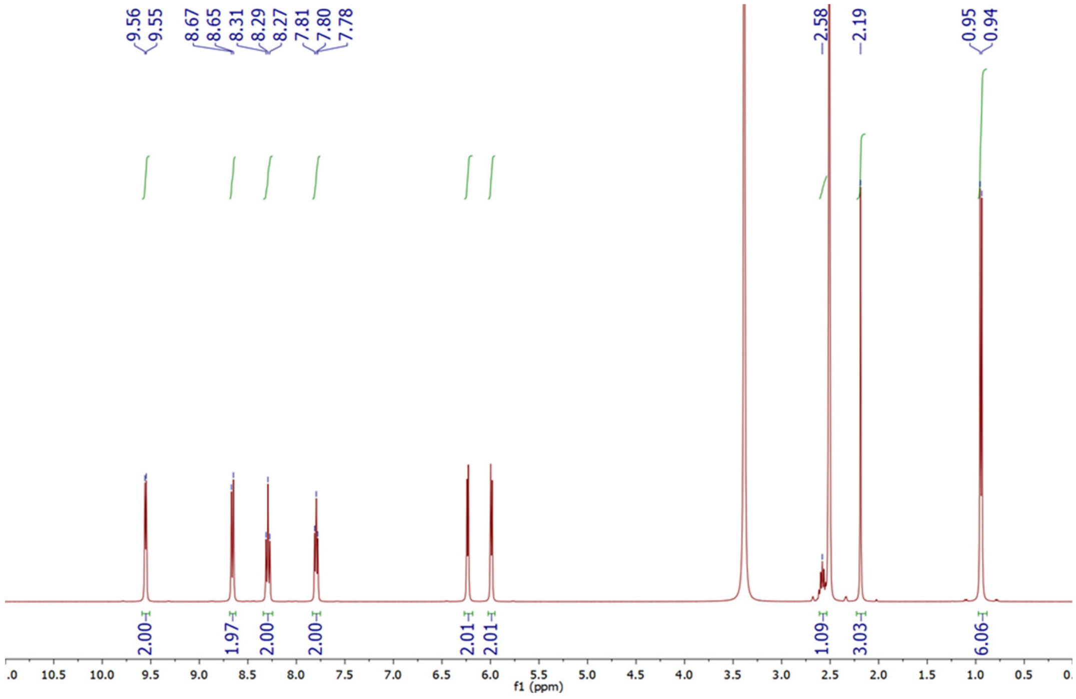

Figure S14. ^{13}C-NMR IC3 in CD_6CO.

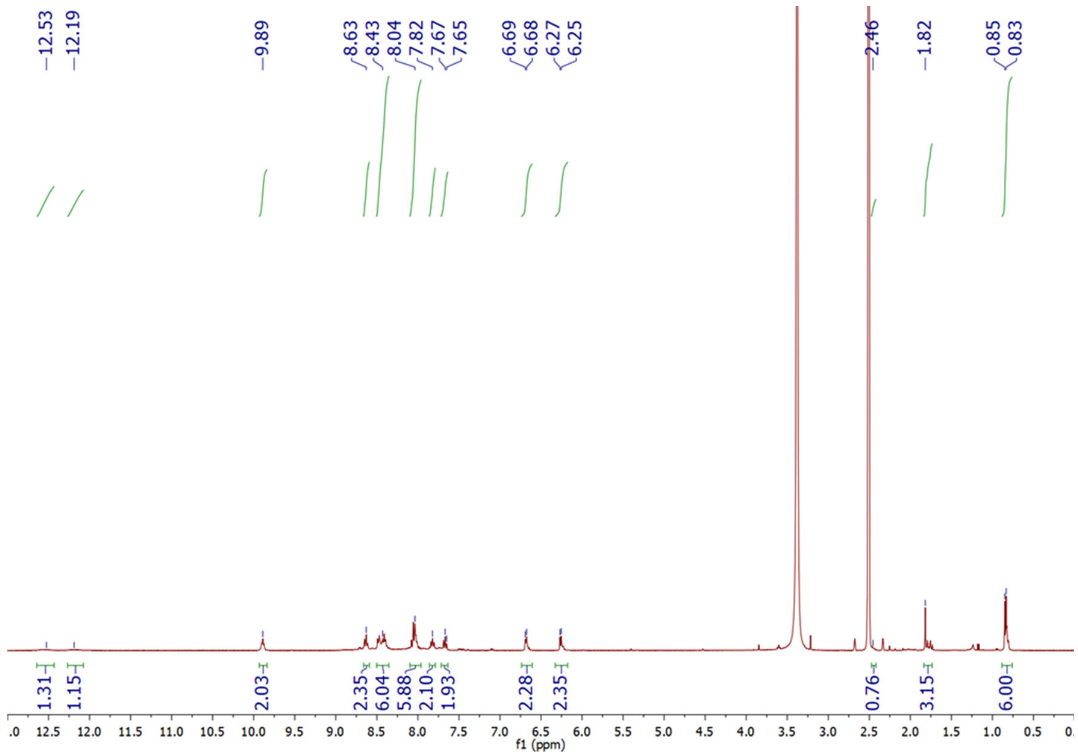

Figure S15. ^{1}H-NMR C in DMSO-D6.

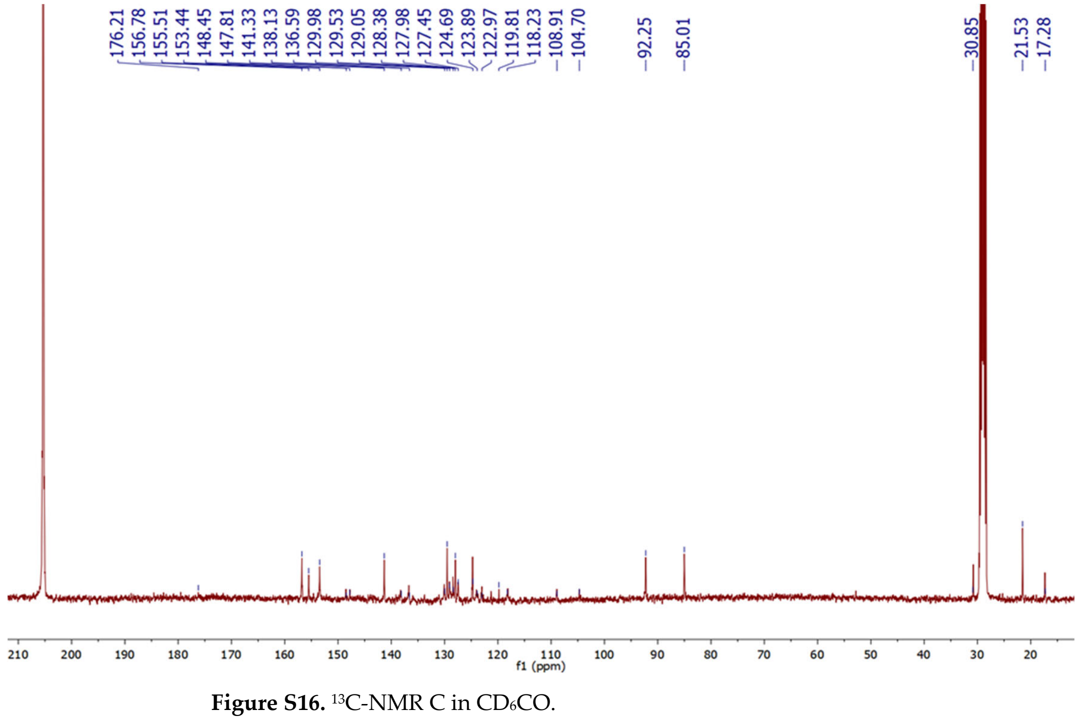

Figure S16. ^{13}C-NMR C in CD6CO.

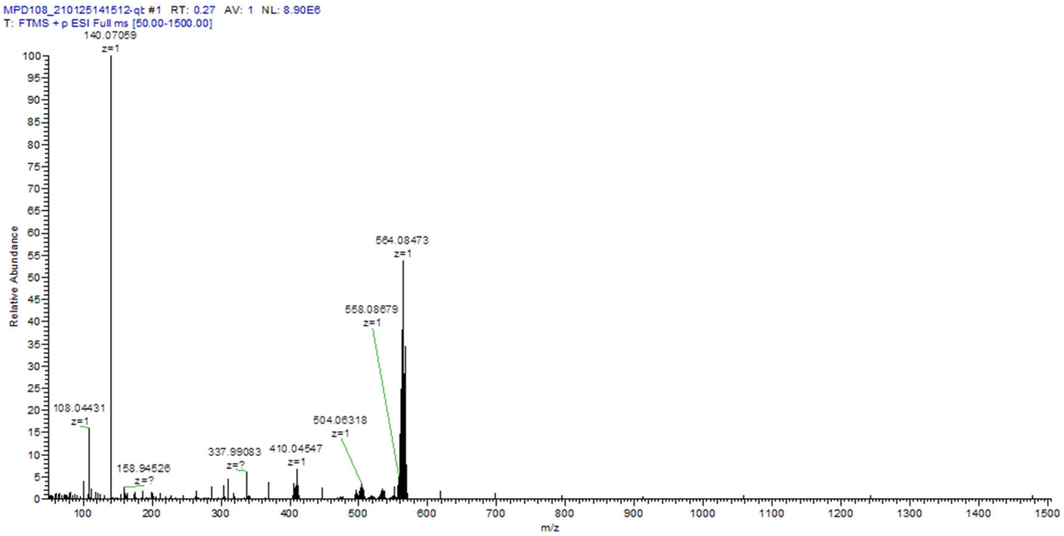

Figure S17. Full HR-MS of IC1. The group of ions above 410 *m*/*z* are induced by the ionization.

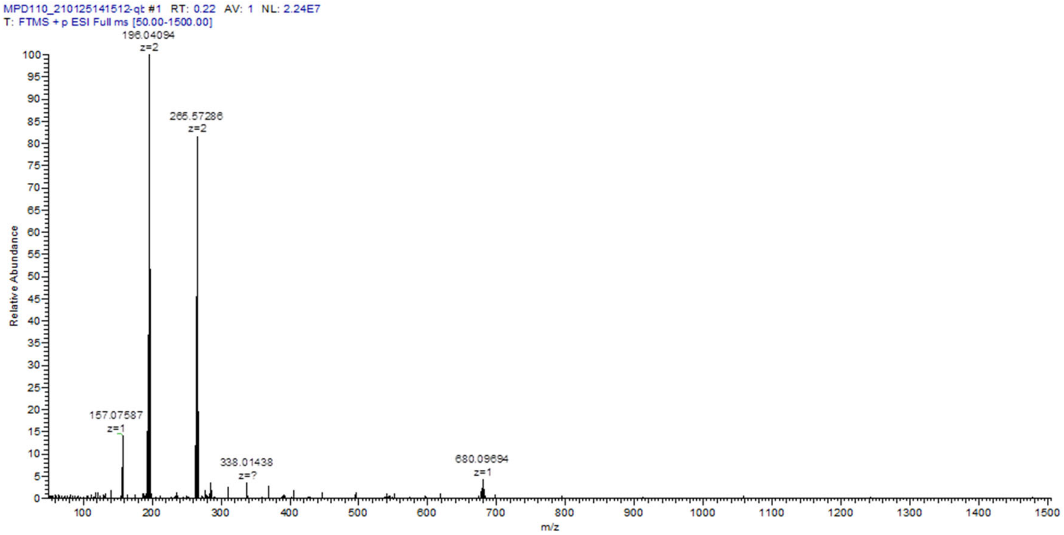

Figure S18. Full HR-MS of IC2.
